# Efficient Obstacle Detection and Tracking Using RGB-D Sensor Data in Dynamic Environments for Robotic Applications

**DOI:** 10.3390/s22176537

**Published:** 2022-08-30

**Authors:** Arindam Saha, Bibhas Chandra Dhara, Saiyed Umer, Kulakov Yurii, Jazem Mutared Alanazi, Ahmad Ali AlZubi

**Affiliations:** 1 Department of Information Technology, Jadavpur University, Kolkata 700098, India; 2 Department of Computer Science and Engineering, Aliah University, Kolkata 700156, India; 3 Department of Computer Engineering, National Technical University of Ukraine “Igor Sikorsky Kyiv Polytechnic Institute”, 03056 Kyiv, Ukraine; 4 Computer Science Department, Community College, King Saud University, Riyadh 11437, Saudi Arabia

**Keywords:** obstacle detection, dynamic obstacle estimation, robot, RGB-D, u-depth map, v-depth map

## Abstract

Obstacle detection is an essential task for the autonomous navigation by robots. The task becomes more complex in a dynamic and cluttered environment. In this context, the RGB-D camera sensor is one of the most common devices that provides a quick and reasonable estimation of the environment in the form of RGB and depth images. This work proposes an efficient obstacle detection and tracking method using depth images to facilitate quick dynamic obstacle detection. To achieve early detection of dynamic obstacles and stable estimation of their states, as in previous methods, we applied a u-depth map for obstacle detection. Unlike existing methods, the present method provides dynamic thresholding facilities on the u-depth map to detect obstacles more accurately. Here, we propose a restricted v-depth map technique, using post-processing after the u-depth map processing to obtain a better prediction of the obstacle dimension. We also propose a new algorithm to track obstacles until they are within the field of view (FOV). We evaluate the performance of the proposed system on different kinds of data sets. The proposed method outperformed the vision-based state-of-the-art (SoA) methods in terms of state estimation of dynamic obstacles and execution time.

## 1. Introduction

Obstacle detection is an active research area for its applicability to autonomous driving or navigation. The objectives are to accurately detect obstacles within the FOV of sensors mounted on robots, measure the obstacle states (i.e., static or dynamic, obstacle dimensions, and the velocities of dynamic obstacles), and predict their future locations to plan for collision-free navigation. An obstacle is nothing but any object that can obstruct the motion of a mobile robot, including an Autonomous Ground Vehicle (AGV) or an Unmanned Aerial Vehicle (UAV). Therefore, an obstacle can be static or dynamic based on its motion. It can have any shape and size, and a dynamic obstacle can also change shape and size dynamically. These characteristics make the task of detecting multiple obstacle and tracking them in a cluttered environment even more complex. A mobile robot may very often requires understanding and tracking of multiple dynamic obstacles and responding quickly to avoid any probable collision. Therefore, the entire process must be onboard to avoid any communication delays. The obstacle detection and processing should also be accurate and less computation-intensive for real-time onboard execution. However, existing approaches are still not adequate for handling all the characteristics that an obstacle may have.

**Figure 1:**
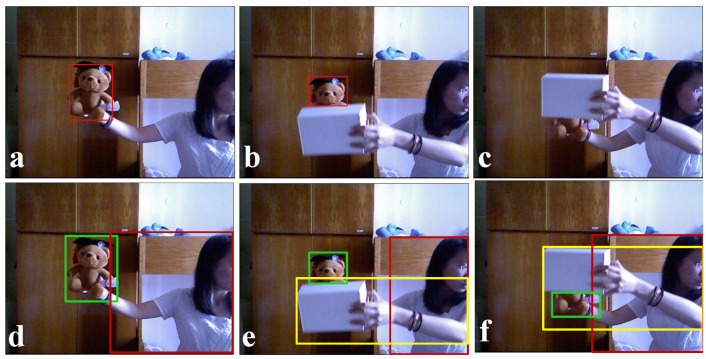
Three snapshots (non-consecutive) were taken from the PTB [1] data set, where (**a**–**c**) show the ground truth of the object (here, the toy bear) tracking marked by red bounding boxes, and (**d**–**f**) show the probable result of any obstacle(s) detection (marked by red, yellow, and green boxes) method.

As with obstacle detection, visual object tracking (VOT) is a similar type of research topic in computer vision. The basic aim of VOT is to track one or more given objects of interest in a given video sequence. The concept of obstacle tracking is a little different from conventional VOT. Let us understand the differences with an example. In Figure 1, three snapshots (non-consecutive) are taken from the PTB data set [1]. The results of the object tracking are shown in Figure 1a–c, where the object of interest is a toy bear and the detected portions of the bear are marked by red bounding boxes. In Figure 1a, the bear is detected. In Figure 1b, the lower portion of the bear is occluded by a box, and hence only the upper portion of the bear is detected, while in Figure 1c, the major portion (face) of the bear is occluded, and therefore VOT failed to detect the object. As we mentioned earlier, obstacle detection means detecting any object that is in front of the capturing device and may obstruct the motion of the robot. Therefore, in obstacle detection, the main target is to detect all the objects. There is no specific object of interest; rather, all objects are objects of interest. Figure 1d–f depicts the possible outputs of an obstacle detection method. In Figure 1d, two obstacles, the bear and the lady, are detected and marked in red and yellow boxes. Figure 1e,f shows three obstacles in red, yellow, and green boxes. These contextual differences limit the usage of VOT directly for obstacle detection and tracking in a robotic environment for navigation purposes.

The rest of this article is organized as follows. In Section 2, we discuss the existing obstacle detection and tracking systems and their limitations. We also highlight our present work in this section. The architecture of the proposed system and our contributions are described in Section 3. A series of evaluations and real-world tests is presented in Section 4. Finally, the conclusions are drawn in Section 5.

## 2. Literature Survey and Motivation of the Work

Existing approaches use both active and passive sensors. Active sensors such as ultrasonic ones are widely used for dynamic obstacle detection, but such sensors have their limitations [2,3], and we restricted our discussion to vision sensors.

In obstacle detection, researchers widely use stereo cameras to calculate the distance of obstacles through disparity measurements from image pairs. Researchers have provided an intense focus on disparity processing on AGV for obstacle detection. Labayrade et al. [4,5] first introduced u-disparity and v-disparity image formation from a stereo disparity and explained their properties for object identification after ground plane segmentation. The work focuses on detecting obstacles that stand on the ground plane, such as other vehicles, trees, and pedestrians. Helen et al. [6] presented a low-latency obstacle avoidance system that uses u-disparity for fast obstacle detection in cluttered environments. Still, dynamic obstacles were kept out of the scope. Kormann et al. [7] showed improved road segmentation with a spline road model and obstacle detection from uv-disparity. Adrian et al. [8] showed multiple representations of the disparity image and uvθ-disparity to achieve obstacle detection. Song et al. [9] presented obstacle detection using a considerate uv-disparity that uses a refined v-disparity for accurate road segmentation. These approaches are equally applicable in a system that helps visually impaired persons [10] because of the similarities in the motion characteristics between humans and AGVs. Therefore, they are limited to AGV-type motion.

Huang et al. [11] presented an obstacle detection system for indoors using Microsoft Kinect [12] that first denoises the depth image using a morphological operation and afterward segments the ground using a v-depth map. The system considers all leftover regions as obstacles after ground segmentation. The region-growing algorithm tracks dynamic obstacles on subsequent frames. The system limits indoor UGV-type motion and requires high computations for denoising and region-growing operations. Lately, low-cost RGB-D cameras (e.g., RealSense D400 [13]) have gained popularity due to technological advancements in terms of indoor and outdoor capabilities, weight reduction, and form factor. These small, lightweight cameras are perfect for fitting on Micro Aerial Vehicles (MAVs). Therefore, researchers focus on detecting objects and obstacles with RGB-D cameras.

In object tracking scenarios, VOT has gained high popularity and has achieved significant progress in the last decade [14,15,16], where some conventional methods mainly focus on tracking objects in RGB video sequences. Despite the considerable advancements, RGB-based tracking faces challenges due to some challenging factors such as a cluttered background, occlusion, and deformation, and researchers explored VOT using RGB-D data. Hannuna et al. proposed a real-time RGB-D tracker, DS-KCF [17], which is built upon the KCF tracker [18] and uses depth cues to handle occlusion, scale variation, and shape changes. Kart et al. proposed CSR-rgbd++ [19], a general framework that uses depth segmentation-based occlusion detection in a discriminative correlation filter framework. Liu et al. proposed a three-dimensional extension of the classical mean-shift tracker [20] that deals with occlusions more effectively. Recently, Kart et al. also proposed OTR, a long term RGB-D tracker that proposes modeling appearance changes via 3D target reconstruction. More recently, Qian et al. proposed DAL [21], an RGB-D tracker that embeds depth information into deep features through the reformulation of a deep discriminative correlation filter (DCF). Very recently, Yan et al. proposed the first offline trained RGB-D tracker, DeT [22], which is based on two RGB trackers: ATOM [23] and DiMP [24]. It uses an additional depth branch to extract depth features and a module for feature fusion. The system is trained using generated RGB-D videos from existing monocular RGB tracking training data. The system also uses the DepthTrack [22] data set for training and showed remarkable performance on the testing set of DepthTrack. These RGB-D trackers are mainly trained using deep networks and require a good amount of contextual data for the training. We excluded these RGB-D trackers from the SoA comparison due to the contextual differences, as shown in Figure 1 and explained in Section 1.

Yang et al. [25] presented a system for dynamic obstacle segmentation that converts a depth image to a point cloud, segments out the planar road, and considers all leftover points as obstacles. The system differentiates static and dynamic obstacles using the DECOLOR algorithm. The system is unsuitable for MAVs because it requires high computations to process the point cloud. Odelga et al. [26] presented an obstacle detection and tracking system for teleoperated UAVs that uses a bin occupancy filter that breaks the entire visible region into smaller bins and searches for the presence of an obstacle in a bin in a probabilistic way. Luiten et al. [27] presented an approach, MOTSFusion, that uses 3D tracklets to estimate dynamic obstacles and their trajectories. The algorithm requires high computation for dense optical flow calculations. Lin et al. [28] presented a vision-based dynamic obstacle avoidance system for MAV (VbOAD). The system uses u-depth maps for detecting obstacles, a multivariate Gaussian probability density function to track obstacles in subsequent frames, and a Kalman filter-based approach to predict their probable future positions and velocities. Estimation of an obstacle’s dimensions using only u-depth maps can become incorrect, as explained in Section 3.2.2. The system assumes a fixed size of obstacles, which is a very constrained assumption that does not hold for real-world scenarios, as captured in the OpenLORIS-Scene market data sequence [29]. The system uses a predefined obstacle height while detecting obstacles from a u-depth map, limiting the system to a predefined obstacle size. The experimental results are limited to only detecting multiple walking humans in an empty room and corridor.

We present a dynamic obstacle detection system aiming to run on board any autonomous system, including UGVs and MAVs, and alleviate some of the problems mentioned above. Our system uses u-depth and v-depth maps for obstacle detection, and we present a new algorithm to track obstacles in subsequent frames. The proposed system estimates the relative velocities between the camera and obstacles and transforms them to the fixed world coordinate frame using self-localization (${}^{w}T_{b}$, *w*, and *b* represent the world and body coordinate frames, respectively). The main contributions of the proposed system are listed below:

- We use dynamic binary thresholding on u-depth maps to improve obstacle detection and their accurate dimension estimation;
- We use restricted v-depth map representation for accurate estimation of obstacle dimensions;
- We present an algorithm for obstacle tracking using efficient processing of u-depth maps;
- The performance of the proposed system on different data sets establishes that the proposed system can detect and estimate the states of multiple static and dynamic obstacles more accurately and faster than SoA algorithms.

## 3. System Architecture

**Figure 2:**
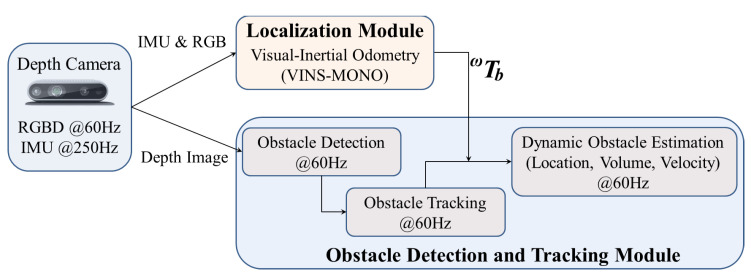
Block diagram of the proposed system for robust dynamic obstacle detection and tracking using RGB-D camera sensor data.

The implementation of the proposed system has two main modules, which are shown in Figure 2. The first module is self-localization, and the second one is dynamic obstacle detection and its tracking. The proposed system can be considered the perception module of any autonomous vehicle, where the primary responsibility of the perception module is to perceive the environment for autonomous navigation. We use the state-of-the-art visual-inertial system VINS-MONO [30] for self-localization. The obstacle detection module receives depth images from any RGB-D sensor, such as the Intel RealSense D435i [13] or Microsoft Kinect [12], where we assume the depth images are rectified, and the obstacle detection and tracking module processes only depth images. Multiple figures in this article show the detected obstacles using our proposed method on multiple RGB images, where those RGB images are used only for better visualization. Finally, the system estimates the velocities of all dynamic obstacles in the world coordinate frame *w*.

### 3.1. Self Localization

The proposed obstacle detection module has the requirements of identifying an obstacle’s state (i.e., static or dynamic) and estimates the velocities of all dynamic obstacles. The system is intended for a mobile robot, and therefore, a static obstacle shows a displacement on consecutive images when the robot is in motion. This means that estimating the motion of any obstacle from the camera coordinate frame is not possible, as the camera frame moves along with the robot. Therefore, we require a fixed coordinate frame for estimating the motions of all obstacles. Any localization module produces robot poses at every instance from a fixed coordinate frame: the world coordinate frame *w*. We can transform the estimated location of any obstacle from the camera coordinate frame to the world coordinate frame with a coordinate transformation through the robot poses. VINS-MONO [30] is one popular SoA localization system that uses monocular images and Inertial Measurement Unit (IMU) measurements to estimate a robot poses in the fixed world coordinate frame *w*. VINS-MONO is open-source software and produces acceptable outcomes in many open sequences, which led us to select VINS-MONO as the localization module in our framework. We refer to [30] for a detailed description of VINS-MONO. If we have a robot pose as ${}^{w}T_{b}$ and a fixed transformation ${}^{b}T_{c}$ from the camera coordinate frame to the body coordinate frame, then $\left({}^{w}T_{b}{}^{b}T_{c}\right)$ is the transformation to transfer the obstacle estimation from the camera coordinate frame to the world coordinate frame.

### 3.2. Obstacle Detection

In this section, we present the obstacle detection part of the proposed system. The input of the obstacle detection module is depth images from the input sensor. VbOAD [28] uses a column-wise histogram representation of the depth image called the u-depth map [4] for obstacle detection and its dimension estimation. The approach in VbOAD has a serious limitation in obstacle height estimation, where empty space is represented as being occupied in a specific situation. Section 3.2.2 explains the limitations of VbOAD with examples. In the proposed method to overcome this limitation, we use two-step depth map representations, where we use the u-depth map representation first. Afterward, we use a restricted row-wise histogram representation of the depth image called a restricted v-depth map.

#### 3.2.1. Depth Map Processing

The objective of depth map processing is to identify obstacles and estimate the positions and dimensions of those obstacles. The obstacle identification and estimation of their attributes are easier from a u-depth map representation because only the specific location of a u-depth map, where obstacles are present, becomes bright horizontal lines that are easy to identify. Therefore, we first discuss the computation of the u-depth map. The u-depth map is a column-wise histogram representing the depth values of the depth image. In this paper, we use row-column order notation to represent any matrix, 2D coordinates, image resolution, or rectangle size. Let us consider a depth image $IMG_{d}$ of a size (H×W), where *H* is the height and *W* is the width, the total number of histogram bins is *N*, and the sensor depth range is from $Min_{d}$ to $Max_{d}$. Then, the range of each bin is $\frac{\left(Max_{d}-Min_{d}\right)}{N}$, and the resolution of the u-depth map is (N×W). The position of any obstacle is estimated from this column-wise histogram, and for better understanding, the histogram is considered a gray image in which white patches represent obstacles. The details of the u-depth map calculation are summarized in Algorithm 1.


**Algorithm 1: U-Depth Map (*IMG_d_, N, Min_d_, Max_d_, uScale*)**
Input: depth image $IMG_{d_{\left(H\times W\right)}}$, number of bins *N*, sensor depth
 
range [$Min_{d},Max_{d}$], scaling factor $uScale=\frac{255}{H}$
Output: u-depth map $uIMG_{d_{\left(N\times W\right)}}$
Step 1. $uIMG_{d}\leftarrow 0$
Step 2. for i = 1 to *W*
 
 
for j = 1 to *H*
 
  
If $Min_{d}\leq IMG_{d}\left(j,i\right)\leq Max_{d}$
 
 
 
$Index=floor[\left\{\frac{\left(N-1\right)}{\left(Max_{d}-Min_{d}\right)}*\left(IMG_{d}\left(j,i\right)-Min_{d}\right)\right\}+1]$
 
 
 
$uIMG_{d}\left(Index,i\right)=uIMG_{d}\left(Index,i\right)+1$
Step 3. $uIMG_{d}=uIMG_{d}*uScale$
Step 4. Return $uIMG_{d}$


**Figure 3:**
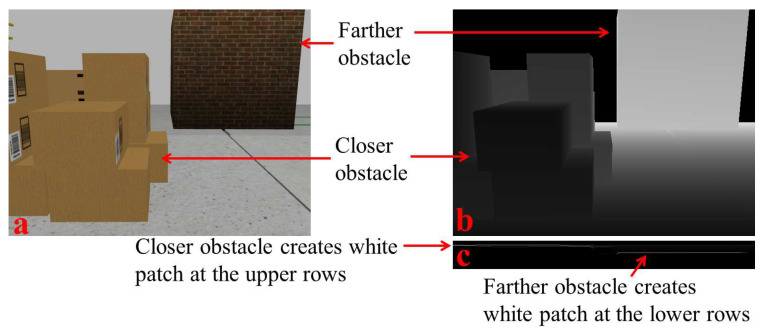
Representation of obstacles in the u-depth map. (**a**) A sample RGB snapshot in a simulated environment under Gazebo [33]. (**b**) Corresponding depth image, where a smaller depth is represented with lower intensity. (**c**) Corresponding u-depth map, in which the white patch position is comparatively at the smaller (or upper) row index for the closer obstacle.

**Figure 4:**
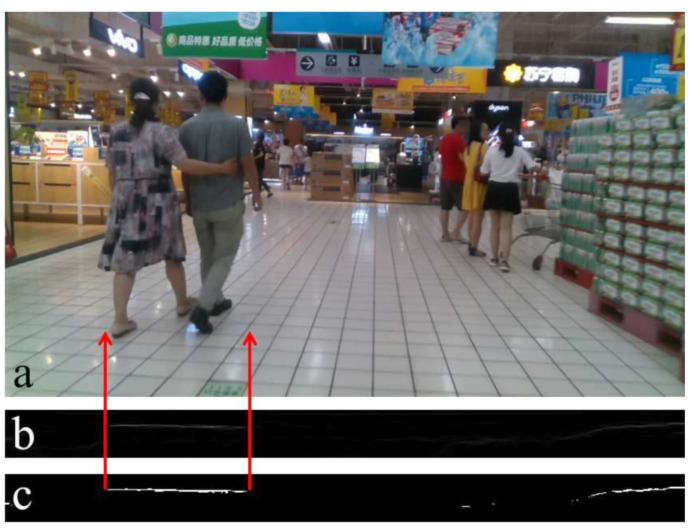
Obstacle’s width estimation from u-depth map. (**a**) Snapshot from OpenLORIS-Scene market data sequence [29]. (**b**) Corresponding u-depth map. (**c**) Corresponding thresholded u-depth map after closing operation.

The above u-depth map calculation differs from the approach in [8], where the u-depth calculation is from a stereo disparity map. In the present method, Step 3 of Algorithm 1 normalizes the values of the u-depth map $uIMG_{d}$∈[0,255] for better processing. Here, the histogram bins in the u-depth map are kept in a growing order from top to bottom (i.e., a row *i* in the u-depth map corresponds to a smaller depth than a row *j* in the u-depth map, where ∀i<j). Therefore, any closer obstacle contributes to the upper rows in the u-depth map. The depth bins corresponding to the obstacles become high and visible as white horizontal patches. Figure 3 depicts a pictorial representation of the relationship between obstacle positions and such white patch positions in a u-depth map with two obstacles positioned in two different depth ranges. The dimensions of a white patch can provide the corresponding obstacle’s position and size in the depth image. Therefore, we require segmenting these white patches in a u-depth map. We compute some basic image processing operations to achieve the required segmentation. First, we convert the u-depth map to a binary image because the white patches become more prominent and easily identifiable. Therefore, we compute binary thresholding where the threshold value is $T_{row}\propto uIMG_{d_{row}}$, which means it is proportional to the distance and provides more important to closer obstacles, even with smaller sizes. This proposed dynamic binary thresholding significantly improves the ability of obstacle detection and accurate dimension estimation. The improvement in obstacle detection is explained with experiments in Section 4.3, and the accuracy improvement in obstacle dimension estimation is explained with the experiments in Section 4.4. The depth estimation of RGB-D sensors such as the InRealSense D435i is noisy. Thus, the u-depth map also contains some noise, making these white patches discontinuous, and very often, a single obstacle can appear as multiple different obstacles. Therefore, we performed a closing operation [31] with a (3×5) structuring element on these white patches and produced continuous patches quickly. Figure 4 shows a u-depth map and its corresponding thresholded u-depth map after the closing operation. Then, we obtain the individual components using the component analysis technique [32]. Hereafter, we use the term ‘u-depth map’ to represent the ‘thresholded and closed binary u-depth map’.

#### 3.2.2. Dimension Calculation

**Figure 5:**
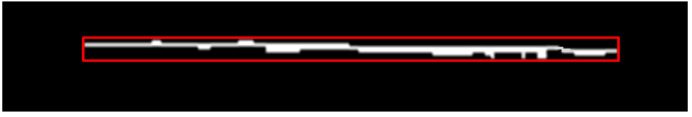
A sample thresholded u-depth map with a segmented obstacle and corresponding bounding box.

In the previous section, we discussed the method for finding the bounding box of an individual obstacle in a u-depth map. Now, we present the method to calculate the dimensions of any obstacle in the 3D world coordinates. First, we obtain the position and dimensions on the depth image (i.e., 2D positions and dimensions), and then we extend the method to find the positions and dimensions in 3D. Let us consider a sample white patch in a u-depth map with a bounding box as shown in Figure 5, where the top-left corner of the bounding box is ($u_{t},u_{l}$) and the right-bottom corner is ($u_{t}+u_{h},u_{l}+u_{w}$). We can find the depth range [$d_{min},d_{max}$] for the corresponding obstacle in the camera coordinate frame (*c*) from the row indexes $u_{t}$ and ($u_{t}+u_{h}$) using Equation (1):

(1) $$\begin{array}{cc} d_{min} & =\left(u_{t}-1\right)*\frac{\left(Max_{d}-Min_{d}\right)}{\left(N-1\right)}+Min_{d}\\ d_{max} & =\left(u_{t}+u_{h}-1\right)*\frac{\left(Max_{d}-Min_{d}\right)}{\left(N-1\right)}+Min_{d} \end{array}$$

**Figure 6:**
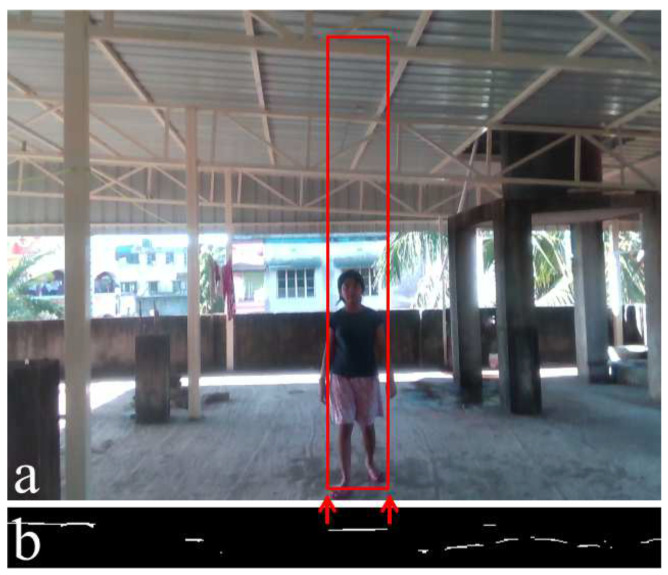
Height estimation of an obstacle using VbOAD [28]. (**a**) A snapshot captured with D435i along with the estimated height of the obstacle (the person). (**b**) Corresponding thresholded u-depth map.

The width of the corresponding obstacle on the depth image will be the same as the width of the white patch on the u-depth map because the u-depth map contains a column-wise histogram. Therefore, the column location does not change from the depth image to the u-depth map calculation. The relation between the width of a white patch and its corresponding obstacle’s width is pictorially shown with red arrows from Figure 4c to Figure 4a. In order to estimate the height of the obstacle, VbOAD [28] selects the minimum and maximum rows in the depth image that contain depth values within the depth range [$d_{min},d_{max}$] and within the column range [$u_{l},\left(u_{l}+u_{w}\right)$]. Now, let us consider a scenario where two or more obstacles are present within the column range [$u_{l},\left(u_{l}+u_{w}\right)$] and within the depth range [$d_{min},d_{max}$]. Then, all those obstacles will contribute to the same depth bins, and a single white patch in the u-depth map will be obtained. Therefore, VbOAD combines the height of all obstacles present within the same column range and the same depth range. Figure 6 shows one such example where the height of a single obstacle becomes the combined height of multiple obstacles. Figure 6a shows a person standing at a certain distance from the camera, and a portion of the roof is also visible at the same distance from the camera. Figure 6b is the corresponding u-depth map, and the red arrows between Figure 6a,b show the width of the person estimated from the white patch of the u-depth map. The estimated height of the person, as in VbOAD, is shown with a red rectangle in Figure 6a. The empty space between the person’s head and the roof becomes a part of the person’s height. In the present method, we tried to resolve this problem. Here, we computed a restricted v-depth map within the column range [$u_{l},\left(u_{l}+u_{w}\right)$] and the depth range [$d_{min},d_{max}$].

**Figure 7:**
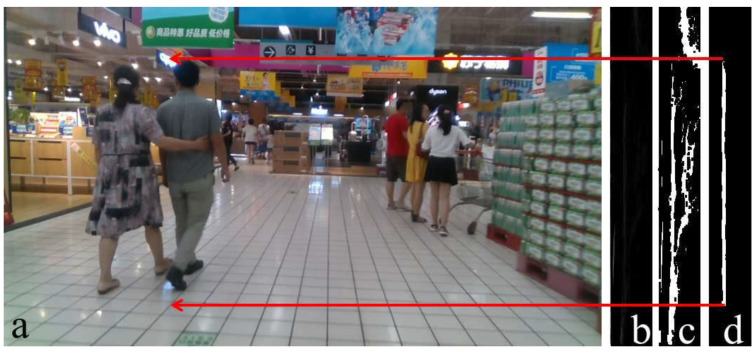
Obstacle’s height calculation using the proposed restricted v-depth map. (**a**) The same snapshot from Figure 4a. (**b**) Corresponding v-depth map. (**c**) Corresponding thresholded v-depth map. (**d**) Corresponding proposed thresholded restricted v-depth map.

We saw that the u-depth map is the column-wise histogram of the depth image. It contains the features to find obstacles and their widths, but cannot produce the correct height of those obstacles in some specific situations, as shown in Figure 6. Similarly, we can take a row-wise histogram of the depth image, and then we should get a vertical white patch for an obstacle, and the height of the obstacle will equal the height of the corresponding vertical patch. The depth of the ground plane grows from bottom to top. Therefore, when we take a row-wise histogram, the ground plane becomes more prominent, and the identification of an obstacle’s height is not straightforward. Labayrade et al. [4,5] showed a row-wise histogram representation of the depth image, a v-depth map, that helps in ground plane segmentation for any UGV. Figure 7 shows the v-depth map pictorially, where Figure 7b shows the v-depth map of Figure 7a. Figure 7c is the corresponding thresholded v-depth map, where the curved white patch is the representation of the ground plane. The obstacle’s height is not understandable from a v-depth map. This is the major drawback of estimating the height of an obstacle from a v-depth map.

In the present method, instead of considering the entire image, we consider only the column ranges of that particular obstacle. As a result, if we only consider the column range [$u_{l},\left(u_{l}+u_{w}\right)$] and the depth range [$d_{min},d_{max}$] in v-depth map formation, then obstacle height estimation becomes simple and accurate because we only use the pixels that lie on the obstacle, and the corresponding white patch only becomes visible on the thresholded image, as shown in Figure 7d. We call this a restricted v-depth map and use it only for accurate height estimation. The proposed restricted v-depth map uses pixels within the column range [$u_{l},\left(u_{l}+u_{w}\right)$] and the depth range [$d_{min},d_{max}$], which means we essentially select only those pixels that lie on those obstacles which are present within the mentioned column and depth ranges. We compute binary thresholding on the restricted v-depth map for a similar reason, as with the u-depth map. The details of the restricted v-depth map with binary thresholding are summarized in Algorithm 2.


**Algorithm 2: Restricted V-Depth Map (**

$IMG_{d},N,u_{l},u_{w},d_{min},d_{max},Max_{d},Min_{d}$

**)**
Input: depth image $IMG_{d_{\left(H\times W\right)}}$, number of bins *N*,
 column range [$u_{l},\left(u_{l}+u_{w}\right)$], sensor depth range [$Min_{d},Max_{d}$],
 obstacle’s depth range [$d_{min},d_{max}$]
Output: Thresholded v-depth map $vIMG_{d_{\left(H\times N\right)}}$
Step 1. $vIMG_{d}\leftarrow 0$
Step 2. for i = 1 to *H*
 
 
for j = $u_{l}$ to ($u_{l}+u_{w}$)
 
   
If $d_{min}\leq IMG_{d}\left(i,j\right)\leq d_{max}$
 
 
 
$Index=floor[\left\{\frac{\left(N-1\right)}{\left(Max_{d}-Min_{d}\right)}*\left(IMG_{d}\left(i,j\right)-Min_{d}\right)\right\}+1]$
 
 
 
$vIMG_{d}\left(i,Index\right)=1$
Step 3. $vIMG_{d}=vIMG_{d}*255$ Step 4. Return $vIMG_{d}$


One important aspect of the above calculation is selecting the threshold value to be one, because the selected column range and depth range allow us to select only those pixels that lie on an obstacle. We do not want to lose a single pixel that lies on the obstacle. We also perform a closing operation with a (5×3) structuring element to generate continuous vertical white patches. Then, we apply component analysis, such as a u-depth map, to find the bounding boxes. The relationship between the height of the white patch and its corresponding obstacle’s height is pictorially shown with red arrows from Figure 7d to Figure 7a. Hereafter, we use the term ‘restricted v-depth map’ to represent the ‘thresholded and closed binary restricted v-depth map’.

**Figure 8:**
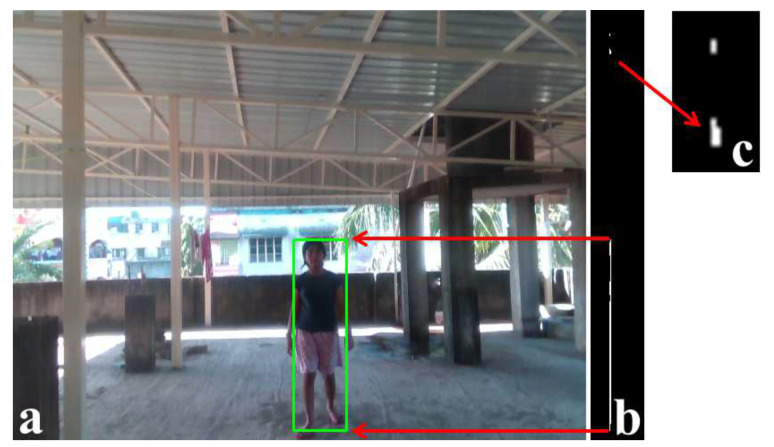
Estimation of the height of an obstacle using the proposed restricted v-depth map. (**a**) Same image used in Figure 6 and the result of the proposed technique. (**b**) Result of the thresholded restricted v-depth map. (**c**) The magnified view of the thresholded restricted v-depth map shows the portion of the roof as a separate obstacle.

The restricted v-depth map produces multiple patches if multiple obstacles exist in the same depth range. Figure 8 shows the height correction of the previous example, where the height of the girl came as the combined height of the girl and the roof, as pictorially shown in Figure 6. Figure 8a shows the height estimation using the proposed restricted v-depth map, and Figure 8b shows the thresholded restricted v-depth map where two different obstacles, the person and a small portion of the roof, are visible. Figure 8c is the magnified view of the portion, where the roof is present in the restricted v-depth map. The estimated height of the person using the proposed restricted v-depth map is shown with a green rectangle in Figure 8a, and the arrows from Figure 8b to Figure 8a show the height of the green rectangle derived from the white patches of the restricted v-depth map.

**Figure 9:**
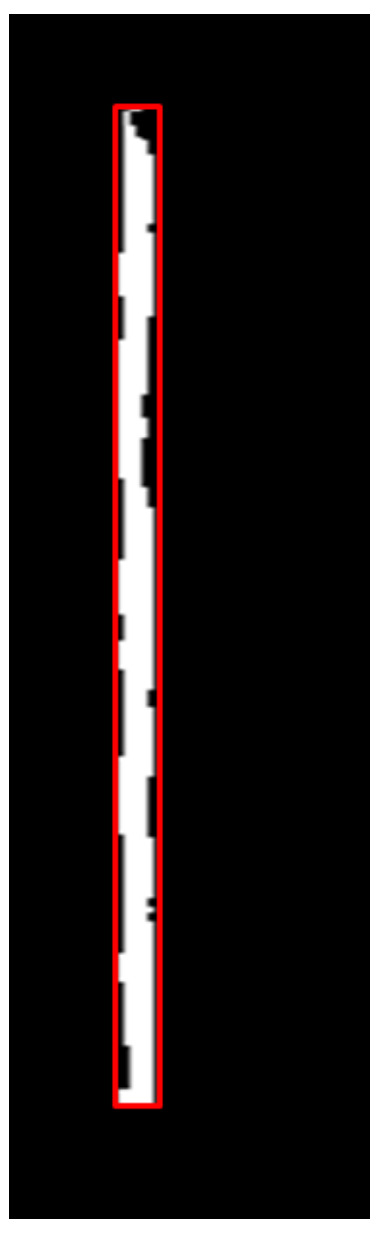
A sample magnified thresholded restricted v-depth map with a segmented obstacle within a bounding box.

Now, let us consider a sample white patch on a restricted v-depth map with a bounding box as shown in Figure 9, where the top-left corner of the bounding box is ($v_{t},v_{l}$) and the right-bottom corner is ($v_{t}+v_{h},v_{l}+v_{w}$). The corresponding obstacle on the depth image must be of the same height as the white patch on the restricted v-depth map. Now, we have the bounding box using the u-depth map represented by coordinates ($u_{t},u_{l}$) and ($u_{t}+u_{h},u_{l}+u_{w}$), and we have the bounding box using the restricted v-depth map with coordinates ($v_{t},v_{l}$) and ($v_{t}+v_{h},v_{l}+v_{w}$). The dimensions of these bounding boxes allow us to calculate the dimensions of the corresponding bounding box on the depth image. Therefore, the bounding box for the current obstacle on the depth image is represented by the coordinates ($v_{t},u_{l}$) and ($v_{t}+v_{h},u_{l}+u_{w}$).

**Figure 10:**
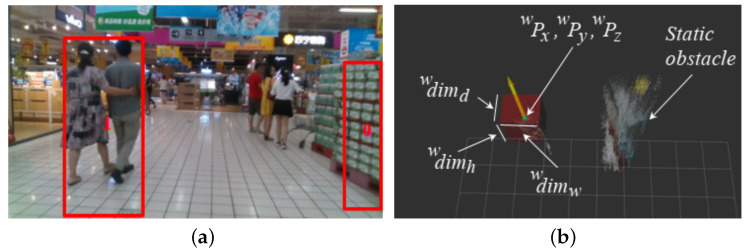
Experimental results of dynamic obstacle tracking with OpenLORIS-Scene market data sequence [29]. (**a**) An RGB image snapshot with all detected obstacles. (**b**) Corresponding Rviz [35] visualization.

We transform this two-dimensional rectangle of the depth image into the three-dimensional camera frame using the relationship between the image plane and the camera frame *c*. We refer to [34] for a detailed description of this relationship. Furthermore, we assume all obstacles are of a rectangular parallelepiped in shape with dimensions (${}^{c}dim_{h}$, ${}^{c}dim_{w}$, ${}^{c}dim_{d}$) and a position (${}^{c}P_{x}$, ${}^{c}P_{y}$, ${}^{c}P_{z}$). Figure 10 presents one example of dynamic obstacle tracking using the proposed method on the OpenLORIS-Scene market data sequence [29], where Figure 10b shows the dimensions of a dynamic obstacle in the world frame. Now, let us consider the most simplistic and popular camera model (i.e., the pinhole camera model) [34], which we use to transform the measurements from two-dimensional to three-dimensional. Here, we take the simplistic form of the intrinsic camera matrix and assume $f_{x}$ and $f_{y}$ are the focal lengths in the image’s horizontal and vertical directions, respectively, and ($c_{y}$, $c_{x}$) is the principal point. Equation (2) first shows the size calculation of the rectangular parallelepiped-shaped obstacle:

(2) $$\begin{array}{cc} {}^{c}dim_{w} & =\frac{\left(\left(u_{l}+u_{w}\right)-c_{x}\right)d_{max}}{f_{x}}-\frac{\left(u_{l}-c_{x}\right)d_{max}}{f_{x}}\\ & =\frac{u_{w}*d_{max}}{f_{x}}\\ {}^{c}dim_{h} & =\frac{\left(\left(v_{t}+v_{h}\right)-c_{y}\right)d_{max}}{f_{y}}-\frac{\left(v_{t}-c_{y}\right)d_{max}}{f_{y}}\\ & =\frac{v_{h}*d_{max}}{f_{y}}\\ {}^{c}dim_{d} & =(d_{max}-d_{min}) \end{array}$$

The expression to calculate the centroid of the rectangular parallelepiped is given in Equation (3):

(3) $$\begin{array}{cc} {}^{c}P_{x} & =\frac{\left(\left(u_{l}+\frac{u_{w}}{2}\right)-c_{x}\right)*\left(d_{min}+\frac{{}^{c}dim_{d}}{2}\right)}{f_{x}}\\ & =\frac{\left(2u_{l}+u_{w}-2c_{x}\right)*\left(2d_{min}+{}^{c}dim_{d}\right)}{4f_{x}}\\ {}^{c}P_{y} & =\frac{\left(\left(v_{t}+\frac{v_{h}}{2}\right)-c_{y}\right)*\left(d_{min}+\frac{{}^{c}dim_{d}}{2}\right)}{f_{y}}\\ & =\frac{\left(2u_{t}+v_{h}-2c_{y}\right)*\left(2d_{min}+{}^{c}dim_{d}\right)}{4f_{y}}\\ {}^{c}P_{z} & =d_{min}+\frac{{}^{c}dim_{d}}{2} \end{array}$$

Let us assume ${}^{b}T_{c}$ is the transformation from the camera coordinate frame to the body coordinate frame. ${}^{b}T_{c}$ is a fixed transformation for any robot, and the estimation of ${}^{b}T_{c}$ is performed offline. We refer to [36] for details on the estimation of ${}^{b}T_{c}$. Let us also assume ${}^{w}T_{b}$ (see Figure 2) is the transformation from the body coordinate frame to the fixed world coordinate frame. The estimation of ${}^{w}T_{b}$ comes from the self-localization module, VINS-MONO [30], as shown in Figure 2. We use the transformation $\left({}^{w}T_{b}*{}^{b}T_{c}\right)$ to transform the rectangular parallelepiped’s location and size from the camera coordinate frame *c* to the fixed world coordinate frame *w*. We estimate the velocities of all obstacles in the world coordinate frame using their temporal movements.

### 3.3. Obstacle Tracking

Tracking associates the detected obstacles in subsequent images and helps predict future positions within a predicted zone. The usual methods of tracking involve associating visual features [25] or using some probability function [28]. Here, we ignore visual features as these are computationally heavy, and the tracking time grows with the size of an obstacle in the image frame. We process a minimal number of pixels from the u-depth map to track obstacles in subsequent frames. One popular way to match two image segments is through Hu Moments [37] calculation, where two images are compared with their associated structural properties. However, the white patches do not have such good structural properties, and we discovered in our multiple experiments that Hu-moment matching produces many false-positive results. Thus, we excluded Hu-moment matching and proposed a suitable matching algorithm.

In this work, we provide a simple matching algorithm. First, we create a signature for an obstacle in the u-depth map and search for a closer signature within a neighboring vicinity in the next frame. Here, we do not consider patches from a restricted v-depth map for the creation of obstacle signatures because we want a fast-tracking algorithm and less complexity. We observed that signatures with the u-depth map were capable of being tracked using our proposed tracking algorithm as described below. Another advantage of using a u-depth map is that if any obstacle moves parallel to the optical direction of the camera (i.e., the relative depth change is at its maximum), the corresponding position change in the u-depth map is minimal, and tracking works well. However, if any obstacle moves from left to right or vice versa, the position change of the obstacle in the u-depth map would be at the same rate as in the depth image, and the tracking algorithm is required to adapt to such movement.

**Figure 11:**
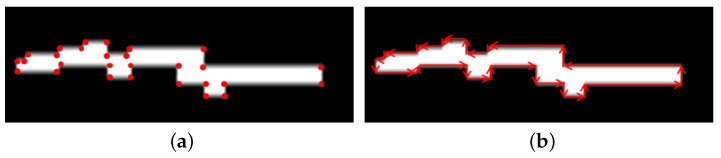
A sample pictorial view of the selected points in $\Phi_{A}$ for the obstacle *A*. (**a**) Red dots are the points present in $\Phi_{A}$. (**b**) Vector formation using the points in $\Phi_{A}$.

Let us assume a sample obstacle *A* consists of a set of white pixels as $P_{A}$ in a u-depth map. Let us consider Figure 11, which shall explain our pixel selection pictorially, where Figure 11a shows the selected white pixels of $P_{A}$. There is another set of pixels $C_{A}\subset P_{A}$ that represents the contour of *A*, where the contour is the ordered set of pixels where two consecutive pixels are neighboring pixels. Now, we create another set that consists of the minimum number of ordered pixel points $\Phi_{A}=\left\{\phi_{1},\phi_{2},\cdots ,\phi_{k}\right\}\subset C_{A}$ such that $\phi_{i}\to \phi_{i+1}\in \Phi_{A}$ for i∈{1,2,⋯,k−1,k} ($\phi_{k+1}\equiv \phi_{1}$) should be connected with a vector ${\hat{l}}_{i,i+1}$ such that the vector completely passes through $C_{A}$. The length of each vector should be as long as possible because the set $\Phi_{A}$ is formed with the minimum number of pixel points. The red dots in Figure 11a represent the points of $\Phi_{A}$, where $|\Phi_{A}|$ is significantly shortened from $|P_{A}|$. Figure 11b shows the vectors that are formed with the points of $\Phi_{A}$. The directions of these vectors depend on the start and end point coordinates. Therefore, they can be in any direction, and Figure 11b also shows that some small vectors are slanted. We compute the extreme left point, $\Phi_{A_{L}}$, and the extreme right point, $\Phi_{A_{R}}$, of $\Phi_{A}$. We also deduce the visibility of the obstacle $Vis_{A}$ in terms of being fully visible or partially visible using the points $\Phi_{A_{L}}$ and $\Phi_{A_{R}}$. We consider obstacle *A* to be partially visible if the point $\Phi_{A_{L}}$ touches the left edge or the point $\Phi_{A_{R}}$ touches the right edge of the u-depth map. We consider {$\Phi_{A}$, $\Phi_{A_{L}}$, $\Phi_{A_{R}}$, $Vis_{A}$} to be the signature of obstacle *A*, where $\Phi_{A}$ contains very few points but retains complete structural information and aids in fast execution. We also create a probable zone around the obstacle *A*. The probable zone selection is based on the maximum allowable relative speed of any obstacle, the FOV and fequency of the input RGB-D camera, and the sensor’s operating depth range.

To match with another obstacle *B* found within the probable zone of obstacle *A* in the next depth image, we first align obstacle *B* with obstacle *A*. The alignment is based on the visibilities of obstacles *A* and *B*. The alignment is at the nearest extreme points (i.e., either {$\Phi_{A_{L}}$, $\Phi_{B_{L}}$}, or {$\Phi_{A_{R}}$, $\Phi_{B_{R}}$}) when both obstacles *A* and *B* are fully visible. The alignment is at the right extreme points (i.e., {$\Phi_{A_{R}}$, $\Phi_{B_{R}}$}) when either of the obstacles is partially visible to the left edge of the u-depth map and vice versa. Equation (4) shows the left and right alignment calculations:

(4) $$\begin{array}{cc} Align_{L_{\left\{A,B\right\}}} & =\Phi_{B_{L}}-\Phi_{A_{L}}\\ Align_{R_{\left\{A,B\right\}}} & =\Phi_{B_{R}}-\Phi_{A_{R}} \end{array}$$

We use either $Align_{L_{\left\{A,B\right\}}}$ or $Align_{R_{\left\{A,B\right\}}}$ to transform the signature of *B* based on the visibility of obstacle *B*, and we name the selected alignment $Align_{\left\{A,B\right\}}$. We measure the dissimilarity between the signature of obstacle *A* and the transformed *B* using Equation (5):

(5) $$Difference_{\left\{A,B\right\}}=Dim_{\left\{A,B\right\}}+Pos_{\left\{A,B\right\}}+Length_{\left\{A,B\right\}}+Angle_{\left\{A,B\right\}}$$

where $Dim_{\left\{A,B\right\}}$, $Pos_{\left\{A,B\right\}}$, $Length_{\left\{A,B\right\}}$, and $Angle_{\left\{A,B\right\}}$ are the dissimilarity costs of the dimension, position, vector length, and vector direction, respectively, between obstacles *A* and *B*. The expression to calculate $Dim_{\left\{A,B\right\}}$ is given in Equation (6), and the $Pos_{\left\{A,B\right\}}$ is calculated using Equation (7):

(6) $$Dim_{\left\{A,B\right\}}=\frac{|∥\left(\Phi_{A_{R}}-\Phi_{A_{L}}\right)∥-∥\left(\Phi_{B_{R}}-\Phi_{B_{L}}\right)∥|}{∥(\Phi_{A_{R}}-\Phi_{A_{L}})∥}$$

(7) $$Pos_{\left\{A,B\right\}}=\frac{∥Align_{\left\{A,B\right\}}∥}{Max_{h}}$$

where $Max_{h}$ is the maximum horizontal pixel displacement permitted for any obstacle. Equation (8) shows the relation for calculating the $Length_{\left\{A,B\right\}}$:

(8) $$Length_{\left\{A,B\right\}}=\frac{\frac{1}{\left|\Phi_{A}\right|}\sum_{i\in \Phi_{A},j\in \Phi_{B}}|(∥{\hat{l}}_{A_{i}}∥-∥{\hat{l}}_{B_{j}}∥)|}{MaxLengthDiff_{\left\{A,B\right\}}}$$

where ${\hat{l}}_{A_{i}}$ is the vector formed with the *i*th and (i+1)th point in $\Phi_{A}$ and ${\hat{l}}_{B_{j}}$ is the vector formed with the *j*th and (j+1)th point in $\Phi_{B}$. $MaxLengthDiff_{\left\{A,B\right\}}$ is the maximum allowable difference in the vector length between obstacles *A* and *B*. The $Angle_{\left\{A,B\right\}}$ between two obstacles (*A* and *B*) is computed using Equation (9):

(9) $$Angle_{\left\{A,B\right\}}=\frac{\frac{1}{|\Phi_{A}|}\sum_{i\in \Phi_{A},j\in \Phi_{B}}\left({\hat{l}}_{A_{i}}⊙{\hat{l}}_{B_{j}}\right)}{MaxAngleDiff_{\left\{A,B\right\}}}$$

where ⊙ calculates the angle between two vectors and $MaxAngleDiff_{\left\{A,B\right\}}$ is the maximum allowable angle difference between the pair (${\hat{l}}_{A_{i}}$, ${\hat{l}}_{B_{j}}$).

We further consider more conditions in estimating the total dissimilarity cost between obstacles *A* and *B* expressed in Equation (5). These conditions are based on the assumptions that we find obstacle *B* almost in the same position of obstacle *A*, and the dimensions of *A* and *B* are nearly identical. Then, we make the closely matching attributes of Equation (5) zero. The conditions are listed below:

- If at least one of the matching obstacle from obstacles *A* and *B* is fully visible, $Dim_{\left\{A,B\right\}}$ is below a threshold $Th_{Dim_{1}}$, and the horizontal component of $Align_{\left\{A,B\right\}}$ is also below a very small threshold $Th_{Align_{1}}$, then we consider $Pos_{\left\{A,B\right\}}$, $Length_{\left\{A,B\right\}}$, and $Angle_{\left\{A,B\right\}}$ to be zero because this is the case where the obstacle *A* almost stays at its previous position, and the width of the obstacle closely matches even after full visibility in one frame.
- If at least one of the matching obstacle from obstacles *A* and *B* is fully visible, and only $Dim_{\left\{A,B\right\}}$ is below the threshold $Th_{Dim_{1}}$, then we consider $Length_{\left\{A,B\right\}}$ and $Angle_{\left\{A,B\right\}}$ to be zero because this is the case where obstacle *A* moves from its previous position, but the width of the obstacle matches closely even after complete visibility in one frame.
- If at least one of the matching obstacle from obstacles *A* and *B* is fully visible, and only the horizontal component of $Align_{\left\{A,B\right\}}$ is below the threshold $Th_{Align_{1}}$, then we consider $Pos_{\left\{A,B\right\}}$ to be zero because this is the case where the obstacle *A* does not move much, but there is a width change due to partial visibility in one frame.
- If both obstacles *A* and *B* are partially visible, $Dim_{\left\{A,B\right\}}$ is below a threshold $Th_{Dim_{2}}$. and the horizontal component of $Align_{\left\{A,B\right\}}$ is also below a very small threshold $Th_{Align_{2}}$, then we consider $Length_{\left\{A,B\right\}}$ and $Angle_{\left\{A,B\right\}}$ to be zero because this is the case where obstacle *A* almost stays at its previous position, and the widths of the obstacles closely match in partial visibilities.

We consider the signatures of obstacles *A* and *B* to be a match one if the $Difference_{A,B}$ is below a certain threshold $Th_{A,B}$. If we find multiple obstacles with a score below $Th_{A,B}$, we consider the obstacle with the lowest score to be the matched one. Once the signature matches, we update the signature with the latest one. This simplistic approach leads to stable tracking with minimal time.

## 4. Experimental Results

For experimental purposes, we used an NVidia Jetson TX2 embedded computing board to implement and test the proposed method in C++ with the Robot Operating System (ROS) [38] environment. In multiple experiments, we evaluated and analyzed the performance of our proposed method using various data sets, such as indoor and outdoor scenes, multiple static and dynamic obstacles, and fast-moving obstacles. Multiple data sets that we used were broadly of five types, and the detailed configurations of all five types are presented in Table 1.

The $Set_{1}$ data set of Table 1 is a self-captured simulated data set recorded in the rosbag format that contains the continuous RGB-D images, IMU measurements, and ground truth poses of all the robots. The $Set_{2}$ and $Set_{3}$ data sets are RGB-D open video sequences, which have the ground truth of object tracking. The $Set_{4}$ data set is a self-captured real data set with continuous RGB-D images and IMU measurements that is also recorded in the rosbag format. We captured multiple sequences of data under this data set, where all of them were outdoors with direct and shaded sunlight on the obstacles, avoiding direct scorching sunlight to avoid depth corruption. The $Set_{5}$ is another open data set, which is available in rosbag format and contains continuous RGB-D images and IMU measurements, as well as the ground truth self-localization pose of the robot. Section 4.2 presents multiple experiments with the $Set_{1}$, $Set_{2}$, and $Set_{3}$ for measuring the accuracy of the proposed system. Section 4.3.1 shows the efficiency of our proposed system in tracking a non-rigid obstacle that changes its size and shape with the $Set_{4}$. Section 4.3.2 shows the performance of our proposed system in a dynamic environment with multiple obstacles having different dimensions with the $Set_{5}$. Section 4.4 shows the accuracy improvement in the dimension estimation of obstacles using the proposed dynamic thresholding with the $Set_{5}$. Section 4.5 and Section 4.6 both show the performance of our proposed system in tracking multiple dynamic obstacles together with the $Set_{4}$ and $Set_{5}$, respectively. Section 4.7 shows the efficiency of our proposed system in tracking a very fast-moving obstacle with the $Set_{4}$.

In all of our experiments, the obstacle detection and tracking modules jointly took 0.4–0.9 ms for a single obstacle, irrespective of its size in the image frame. The average tracking time was about 4–5 ms, considering a maximum of five obstacles. Therefore, the system can perform in real time with five obstacles or more with a 60-Hz camera. The tracking algorithm confirmed successful tracking of an obstacle with a maximum velocity of 2.5 m/s with a 60-Hz camera.

### 4.1. Parameter Tuning

We set the values of the parameters that were introduced in Section 3.3 experimentally, and each parameter held a fixed value throughout all of our experiments. $Max_{h}$ is the maximum allowable horizontal pixel displacement for any obstacle, which means $Max_{h}$ is related to the obstacle speed and quantified in the pixel domain. Therefore, the value $Max_{h}$ must be higher in the cases where either the environment has fast-moving obstacles or the width of the images is large. Setting $Max_{h}$ to a large number allows the system to deal with fast-moving obstacles, but it increases the processing time. We found setting the value of $Max_{h}$ to 6–7% of the image width produced stable tracking, and we set $Max_{h}$ to 40 for the resolution 480×640 and 49 for the resolution 480×848. $MaxLengthDiff_{\left\{A,B\right\}}$ and $MaxAngleDiff_{\left\{A,B\right\}}$ represent the maximum allowable length and angle differences between two vectors. These values basically encode the changes in the signature of an obstacle in the u-depth map due to the motion. Therefore, keeping lower values for these parameters enforces a hard constraint in signature matching between two obstacles and rejects those with a small signature mismatch. On the other hand, a large value for these parameters can increase the false-positive matching result. In our experimental evaluation, we set $MaxLengthDiff_{\left\{A,B\right\}}$ = 30 pixels and $MaxAngleDiff_{\left\{A,B\right\}}$ = 100${}^{\circ }$. The pair ($Th_{Align_{1}}$, $Th_{Dim_{1}}$) and ($Th_{Align_{2}}$, $Th_{Dim_{2}}$) are two level threshold measurements. Each level considers the alignment distance and dimension dissimilarities between the two signatures, respectively. The first level indicates an exact match, and the second level indicates a good match between the two signatures. The two levels of thresholding are created to use some clues to match quickly between two closely related signatures through bypassing some amount of processing. Therefore, this two-level thresholding concept decreases the total processing time and does not have any effect on the accuracy of the system. The values are experimentally set to $Th_{Dim_{1}}$ = 0.1, $Th_{Dim_{2}}$ = 0.25, $Th_{Align_{1}}$ = 10, and $Th_{Align_{2}}$ = 20. Finally, we accepted a signature as matched if the matching score was below the threshold $Th_{A,B}$, which again was set experimentally to 1.5.

### 4.2. Obstacle Tracking Accuracy

We evaluated the accuracy of the proposed algorithm with the $Set_{1}$, $Set_{2}$, and $Set_{3}$ data sets from Table 1. We first discuss the environmental set-up of the $Set_{1}$ data set and then proceed to discuss the experiments conducted on the $Set_{1}$. A detailed description of the experiments with the $Set_{2}$ and $Set_{3}$ is covered afterward in Section 4.2.5.

#### 4.2.1. Gazebo Environment and Experimental Set-Up

**Figure 12:**
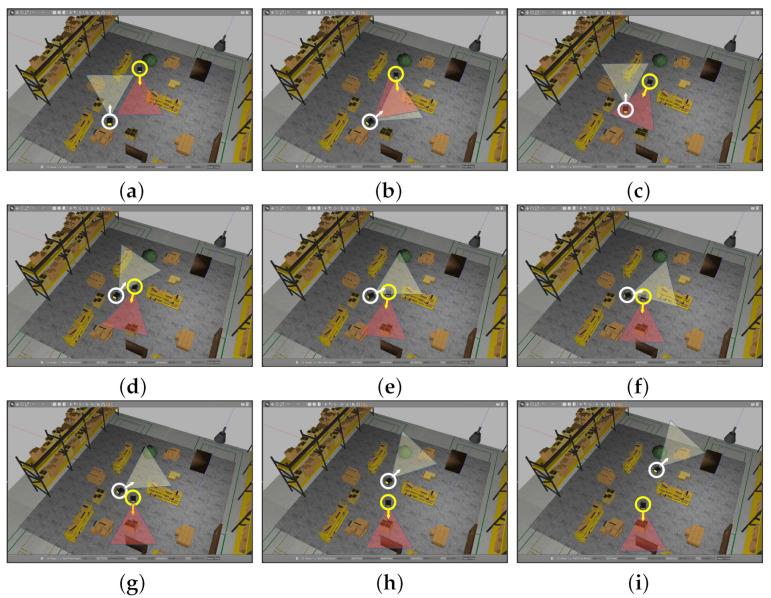
Snapshots of Gazebo [33] environments, where Husky1 is labeled with a white colored circle and Husky2 is labeled with a yellow colored circle. (**a**–**i**) are time-sampled snapshots depicting the motions of Husky1 and Husky2.

We created a shop floor environment with two Husky robots [39], defining paths with a series of way points. The defined path ensured that Husky1 (in Figure 12) watched Husky2 in motion, which made Husky1 view multiple static obstacles and a dynamic obstacle (Husky2) during its motion. Figure 12 presents some snapshots of the environment for a better understanding of their motion pictorially, where two Husky robots are encircled with white and yellow circles, respectively. Their camera viewing directions are shown with arrow lines, and the FOV is shown with colored triangles. The sizes and directions of the arrows and FOV triangles are merely indicative and do not correspond to the actual scales.

We compared the proposed obstacle detection and tracking algorithm with Boosting [40], KCF [18], MedianFlow [41], MIL [42], MOSSE [43], TLD [44], and VbOAD [28]. The first six algorithms use RGB image-based tacking, whereas VbOAD and the proposed method use depth image-based tracking. RGB image-based tracking is highly dependent on the initialization, because these algorithms segment the initialized region of interest as foreground and background and try to track the foreground in subsequent image frames. In real-life scenarios, obstacles usually come from the outside to within the FOV, making them partially visible at the beginning and gradually becoming fully visible once they come completely within the FOV. Therefore, we performed two kinds of testing, where the first one initialized the RGB-based tracking algorithms with partial visibility of Husky2. In contrast, the second one initialized the RGB-based tracking algorithms with full visibility of Husky2. VbOAD and the proposed algorithm detected Husky2 as soon as it was partially visible in both the tests. We used the OpenCV [45] implementation for all RGB-based tracking.

#### 4.2.2. Initialization at Partial Visibility of Husky2

**Figure 13:**
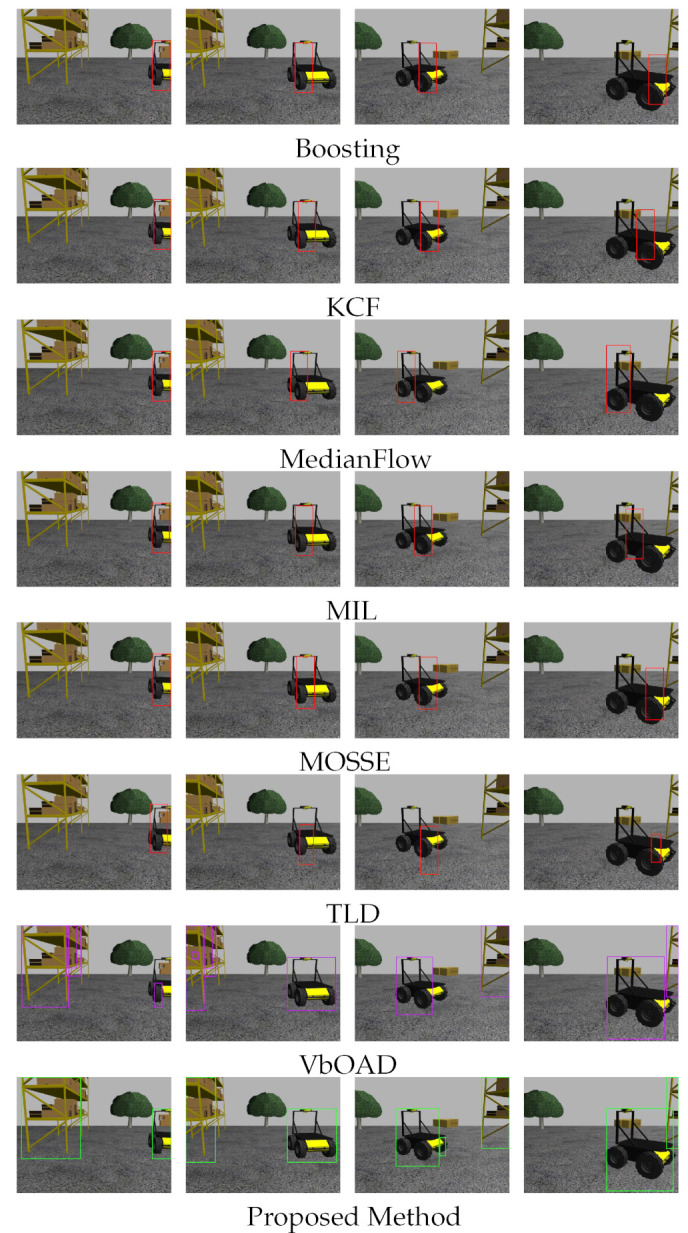
Comparison among the mentioned tracking algorithms with a dynamic obstacle: Husky2. All algorithms were initialized when the Husky2 became partially visible (first column).

In this experiment, all algorithms, as mentioned before, initialized or detected Husky2 as a dynamic obstacle as soon as it became partially visible to Husky1. Figure 13 shows the tracking and dimension estimation results of Husky2, where the detected position and dimensions are drawn with bounding boxes. The first column of Figure 13 represents the initialization frames, where Husky2 is partially visible. The RGB-based tracking algorithms (first six rows in Figure 13) produced erroneous tracking results and erroneous obstacle dimensions when Husky2 became fully visible. The VbOAD algorithm executed with a threshold height of 1 m. It identified Husky2 as soon as it was partially visible in the depth image and generated more accurate results than all RGB-based tracking. The proposed method also detected Husky2 as soon as it appeared partially in the depth image. The estimated dimensions of Husky2 outperformed VbOAD, as shown in the last two snapshots in Figure 13, where the obstacle dimensions were more accurate in the proposed method.

#### 4.2.3. Initialization at Full Visibility of Husky2

**Figure 14:**
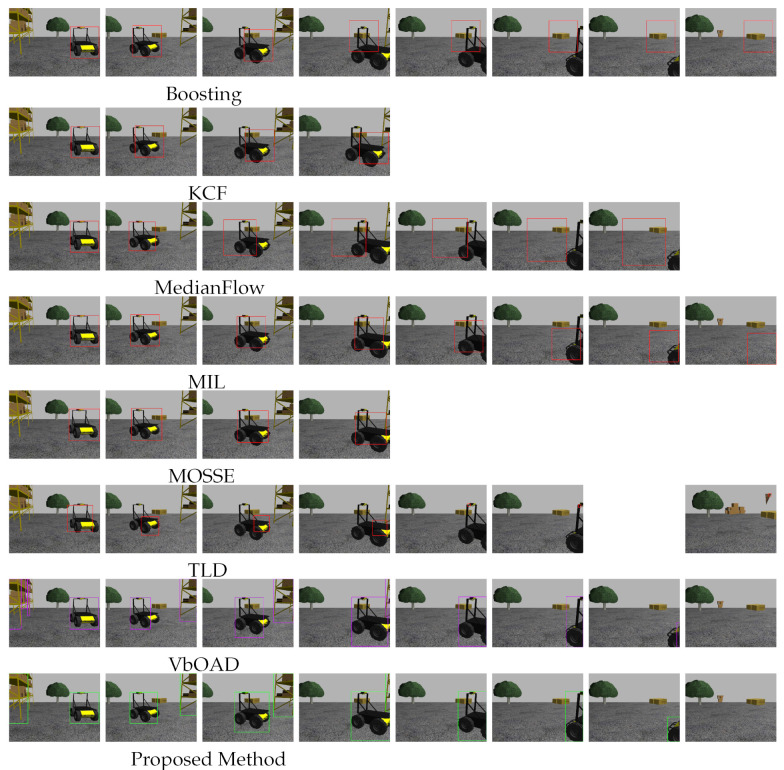
Similar type of experiment to that presented in Section 4.2.2. In this experiment, all RGB-based algorithms (top six rows) were initialized when the Husky2 became completely visible (first column).

In this experiment, all RGB image-based tracking algorithms, as mentioned before, initialized or detected Husky2 as a dynamic obstacle when it became completely visible, but VbOAD and the proposed algorithms detected Husky2 as soon as it was visible partially on the depth image, similar to the experiment in Section 4.2.2. Figure 14 shows the tracking and dimension estimation results of Husky2, where the detected position and dimensions are drawn similar to Figure 13. The first six rows of column one in Figure 14 represent the initialization frames for all RGB image-based tracking algorithms, where Husky2 is completely visible. In this experiment, we found that the performance of KCF and MOSSE was better than other RGB-based tracking algorithms, because these two algorithms could detect when Husky2 was exiting the FOV and stop tracking as soon as Husky2 became partially invisible. The Boosting algorithm produced erroneous results when Husky2 went out of the FOV and became partially invisible, and it kept producing erroneous tracking results even after Husky2 was totally out of the FOV. This is visible in the snapshots of the fourth through eighth columns in row one of Figure 14. The MedianFlow algorithm started producing erroneous results when Husky2 changed its appearance from the initialization appearance, as shown in the third column in row three of Figure 14, and the estimation error increased as Husky2 partially went out of the FOV, which is visible in the snapshots from the fourth through seventh columns in row three. The MedianFlow algorithm also produced erroneous tracking results similar to the Boosting algorithm when Husky2 was totally out of the FOV. We found MIL to be the best algorithm among the RGB-based tracking algorithms we used in this experiment. The performance of the algorithm was similar to the Boosting algorithm. Still, the dimension estimation was less erroneous than the Boosting algorithm, as shown in the snapshots in the fourth row of Figure 14. The TLD algorithm had a more significant influence on the appearance of the obstacles. Therefore, it was more prone to producing erroneous results when an obstacle changed its appearance. We found erroneous tracking results in the dimension estimation of Husky2, as shown in the snapshots from the second through sixth columns in row six of Figure 14. The VbOAD algorithm executed with a threshold height of 1 m and generated more accurate results, as in the previous case in Section 4.2.2. Still, the proposed method outperformed the VbOAD in terms of dimension estimation of Husky2, as visible in the snapshots in the second, third, and seventh columns of row eight in Figure 14.

#### 4.2.4. Accuracy Comparison on $Set_{1}$

**Figure 15:**
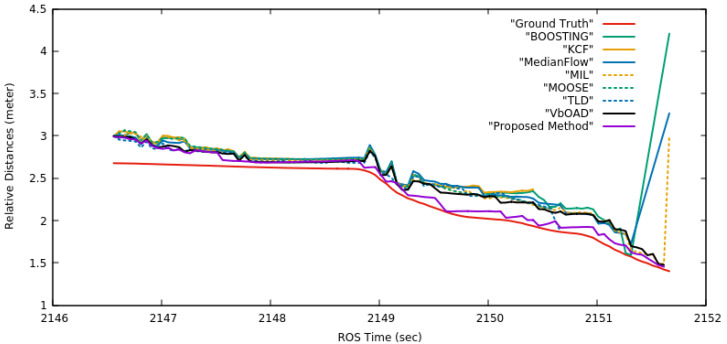
Comparative analysis of deviation of the estimated relative distance with actual relative distance of Husky2 from Husky1. All estimations are in the camera coordinate frame *c*.

We evaluated the accuracy of the proposed method against the ground truth of the experiment presented in Section 4.2.3 and Figure 14. We also evaluated the accuracy of all other SoA algorithms presented in the Section 4.2.3. Figure 15 presents a comparative analysis in terms of the deviation of the estimated relative distances from the actual distances of Husky2 from Husky1. We calculated the ground truth of the relative distances by taking the absolute positions of Husky1 and Husky2 from the Gazebo. These absolute positions were measured in the body coordinate frame *b*. Therefore, this ground truth was the relative distances between the two IMUs of the two Huskies. The ground truth is the red-colored curve in Figure 15, and any estimation curve closer to the ground truth curve is more accurate. All tracking algorithms present in Figure 15 did not have any knowledge about the position of the IMU in Husky2 and calculated the relative position in the camera coordinate frame *c* as explained in Equation (3). The Boosting, MedianFlow, and MIL algorithms showed a sudden jump between 2151 sec and 2152 sec to high distances because these algorithms detected an erroneous obstacle at very far distances after Husky2 went out of the FOV. This incident is captured in the snapshots of the sixth through eighth columns of row one, seventh and eighth columns of row three, and eighth column of row four in Figure 14. The relative distance estimation curve of the proposed method (purple color) is the closest to the ground truth curve among all the SoA methods presented in Figure 15. Therefore, we can conclude that the proposed dynamic obstacle tracking and dimension estimation was the best among all the SoA algorithms presented in this experiment and shown in Figure 15.

**Figure 16:**
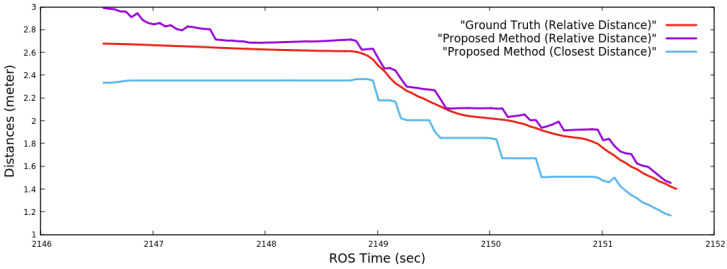
The closest distance estimation of the Husky2 by the proposed method against the ground truth of relative distances of the experiment presented in Section 4.2.3.

Figure 16 compares the estimated closest distances of the proposed method against the ground truth of relative distances. The estimated minimum distance is the distance between the optical center of the Kinect [12] of Husky1 and the closest surface of Husky2, as detected from the depth image. Therefore, for any time instance, the minimum distance should always be smaller than the corresponding relative distance between the IMUs of the two Huskies [39], and this effect is captured in the comparison graph. The closest distance is significant for any obstacle avoidance algorithm for safe, collision-free navigation.

**Figure 17:**
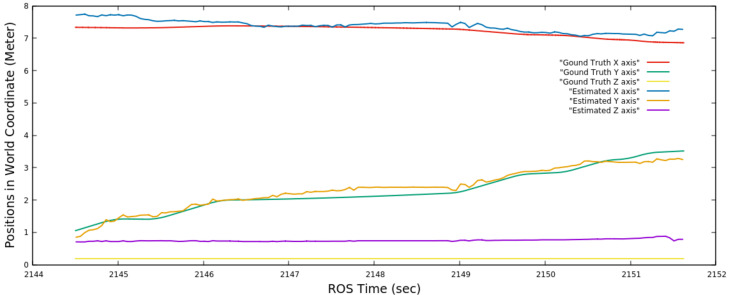
Absolute positional estimation of Husky2 in the world coordinate frame by the proposed method against the ground truth of the experiment presented in Section 4.2.2. The estimation curve closer to the corresponding ground truth curve is more accurate.

Figure 17 shows the estimation of the absolute positions in all three axes in the world coordinate frame against the ground truth. The absolute position of Husky2 was calculated as the centroid of the detected dynamic obstacle using the proposed method. The XY plane was the ground plane in this experimental set-up. Therefore, the estimations on the X and Y axes became better as soon as Husky2 came close to Husky1, and again, the error increased toward the end when Husky2 became partially invisible to Husky1. The Z-axis had an almost constant error, and this error was because the actual IMU position of Husky2 was lower than the estimated centroid. We created 12 motions within this Gazebo environment, where one Husky could see the other in motion and execute each set 5 times. The maximum error we found in all of our evaluation tests was 0.9 m.

#### 4.2.5. Accuracy of the Proposed Method for $Set_{2}$ and $Set_{3}$

**Figure 18:**
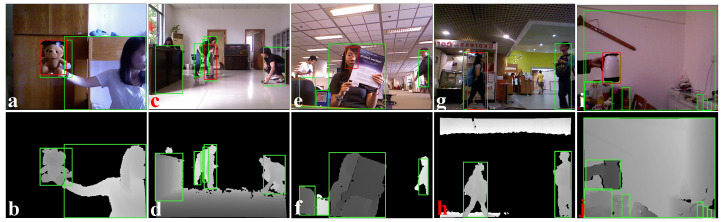
RGB and corresponding depth snapshots of from the PTB data set [1]. (**a**,**b**): bear_front, (**c**,**d**): child_no1, (**e**,**f**): face_occ5, (**g**,**h**): new_ex_occ4, and (**i**,**j**): zcup_move_1. The RGB images show our obstacle estimation in green rectangular boxes, and the ground truth annotations are in red rectangular boxes.

**Figure 19:**
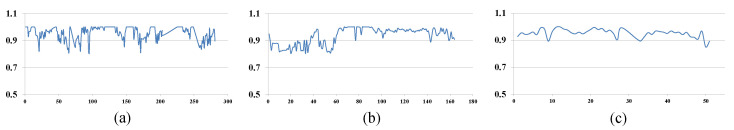
ACC plot of our estimation on (**a**) bear_front, (**b**) child_no1, and (**c**) new_ex_occ4 video sequences of PTB data set [1]. Error value one represents the best estimation.

We evaluated the proposed method with two open data sets: PTB [1] and DepthTrack [22]. Each of these data sets contains multiple RGB-D videos, where the ground truth is provided for object tracking for each video in the form of a list of rectangles and a single rectangle for each frame of the video. These rectangles represent the bounding boxes around a specific object in a video. Our proposed obstacle detection, as explained in Section 3, processes only depth images and does not perform object-level segmentation. Therefore, the conceptual differences between object detection and obstacle detection, as explained in Section 1 and shown in Figure 1, limited our ability to compare our estimation directly with the provided ground truth. In this case, we defined a separate formulation to be compared with the ground truth. Let us consider that $A_{g}$ is the ground truth rectangular area and $A_{e}$ is our estimated rectangular area. Now, we define the accuracy measure as $ACC=\left(A_{e}\cap A_{g}\right)/A_{g}$. ACC=0, representing the worst case, and ACC=1, representing the best case. Figure 18 shows a snapshot from all five training sequences of the PTB data set with our estimations and the ground truth. The depth images (Figure 18b,d,f,h,j) with our estimations provide a better perception of accurate obstacle detection and dimension estimation. Figure 19 shows the accuracy plots of our evaluations on three video sequences of the PTB training data set. The accuracy values were always greater than 0.8. We notice that the accuracy was reduced close to 0.8 whenever multiple obstacles were in close vicinity, and depth discontinuities were not prominent.

**Figure 20:**
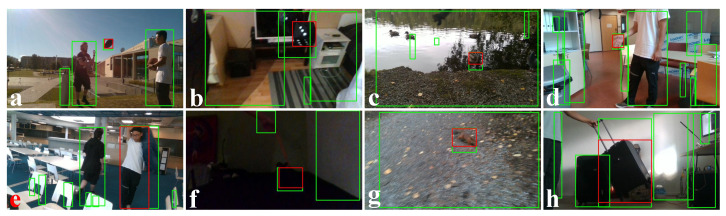
The results of our proposed obstacle tracking system on (**a**) ball10_wild, (**b**) cube03_indoor, (**c**) duck03_wild, (**d**) hand01_indoor, (**e**) human02_indoor, (**f**) pot_indoor, (**g**) squirrel_wild, and (**h**) suitcase_indoor video sequences of DepthTrack data set [22].

Figure 20 shows the tracking results of our proposed method on eight video sequences from the DepthTrack [22] data set. We covered indoor and outdoor lighting, slow and fast motion, and small and big objects within these eight sequences. The system failed to track the obstacle (hand of a person) multiple times in the hand01_indoor video sequence (Figure 20d). The dynamic thresholding on the u-depth map provides more importance to a small obstacle when it is close to the camera, but the obstacle was quite small and far from the camera in the hand01_indoor video sequence, and thus it was rejected. Table 2 contains the average ACC values (i.e., $ACC_{avg}$) of all tested video sequences from the PTB and DepthTrack data sets.

### 4.3. Dynamic Obstacles and Dynamic Size

We evaluate the proposed method with a dynamic obstacle that abruptly changed its shape and size while in motion. We chose this scenario because it is a common behavior where robots and humans work in the same environment, and different human motions result in dynamic obstacles that change their shape and size. Furthermore, we performed two comparison experiments of the proposed method against the VbOAD on two different data sets: the first with the $Set_{4}$ and the second with the $Set_{5}$ of Table 1.

#### 4.3.1. Single Dynamic Obstacle with Varying Height ($Set_{4}$)

**Figure 21:**
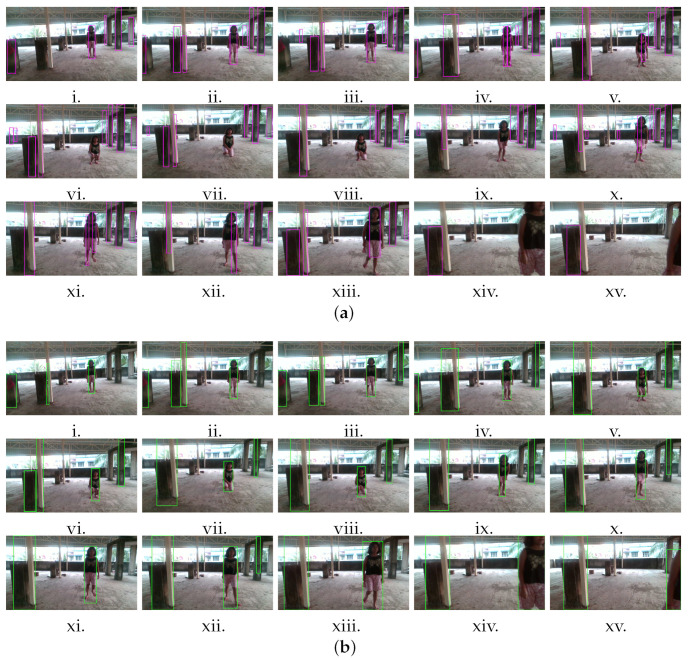
Comparison of the proposed method with VbOAD [28] algorithm with a dynamic obstacle that changed its shape and size abruptly on the run. (**a**) (**i**–**xv**) Time-sampled snapshots with the output of VbOAD. (**b**) (**i**–**xv**) Time-sampled snapshots with the output of our proposed method.

In the first experiment, a girl of a height of 1.53 m walked toward the camera at an average speed of 1 m/s. She bent down suddenly for 4 s, stood up again, and started walking. Figure 21 presents a comparison of the proposed method with the VbOAD. Figure 21a presents the tracking results of the VbOAD, where obstacles are denoted with purple-colored bounding boxes. The VbOAD uses a fixed threshold on the u-depth map, and the threshold value was set to 1.524 m in this experiment. Therefore, the VbOAD detected all obstacles with heights equal to or greater than 1.524 m. The VbOAD algorithm failed to detect any obstacle with a height below the threshold of 1.524 m. As a result, the VbOAD failed to detect the girl when she bent down and shortened her stature, as illustrated in Figure 21a(vi–ix). Figure 21b shows the tracking results of the proposed method. The proposed method successfully detected and tracked the girl until she was within the FOV, as shown in Figure 21b(v–xv).

#### 4.3.2. Multiple Obstacles with Different Heights ($Set_{5}$)

**Figure 22:**
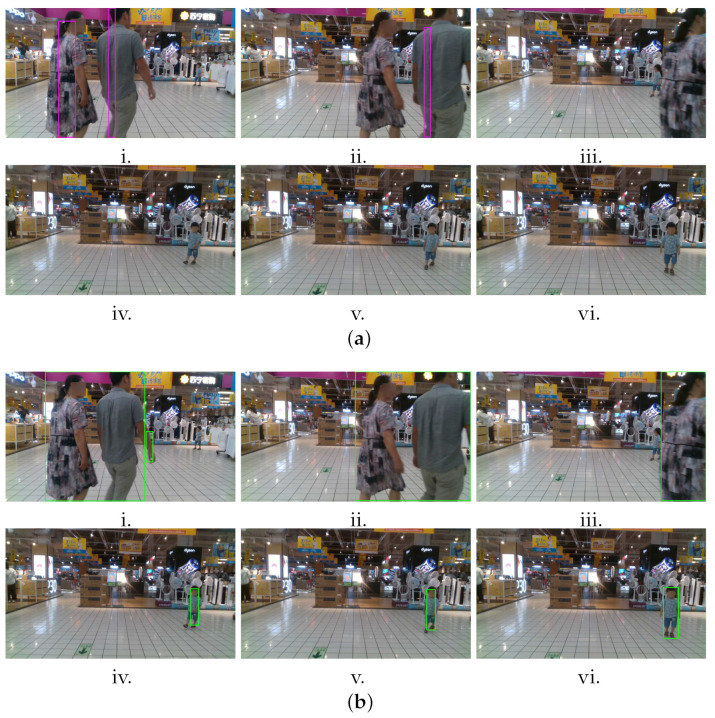
Comparison of the proposed method with VbOAD [28] algorithm on the market sequence of Open LORIS-Scene data set [29] with multiple dynamic obstacles of different sizes. (**a**) (**i**–**vi**) Time-sampled snapshots with the output of VbOAD. (**b**) (**i**–**vi**) Time-sampled snapshots with the output of our proposed method.

In the second experiment, two people were visible toward the left of the image, and they gradually walked toward the right and left the FOV. Afterward, a walking child suddenly came within the FOV from the right edge of the image. Figure 22 presents the comparison output between VbOAD and the proposed method. Figure 22a shows the snapshots of the tracking algorithm of VbOAD, where obstacles are denoted with a purple-colored bounding box, and Figure 22b shows the snapshots of the tracking algorithm of the proposed method, where obstacles are denoted with a green-colored bounding box. The VbOAD algorithm executed with a 1.524-m obstacle height, successfully detecting the two walking persons. Still, it failed to identify the true dimensions of the obstacles because of wrong thresholding, as shown in Figure 22a(i,ii). The VbOAD algorithm failed to detect the small child of the height of about 1 m, as shown in the snapshots in Figure 22a(iv–vi). The proposed dynamic thresholding on the u-depth map successfully detected and estimated the dimensions of obstacles of various sizes, as shown in Figure 22b.

### 4.4. Accuracy Improvement with Dynamic U-Depth Thresholding on $Set_{5}$

**Figure 23:**
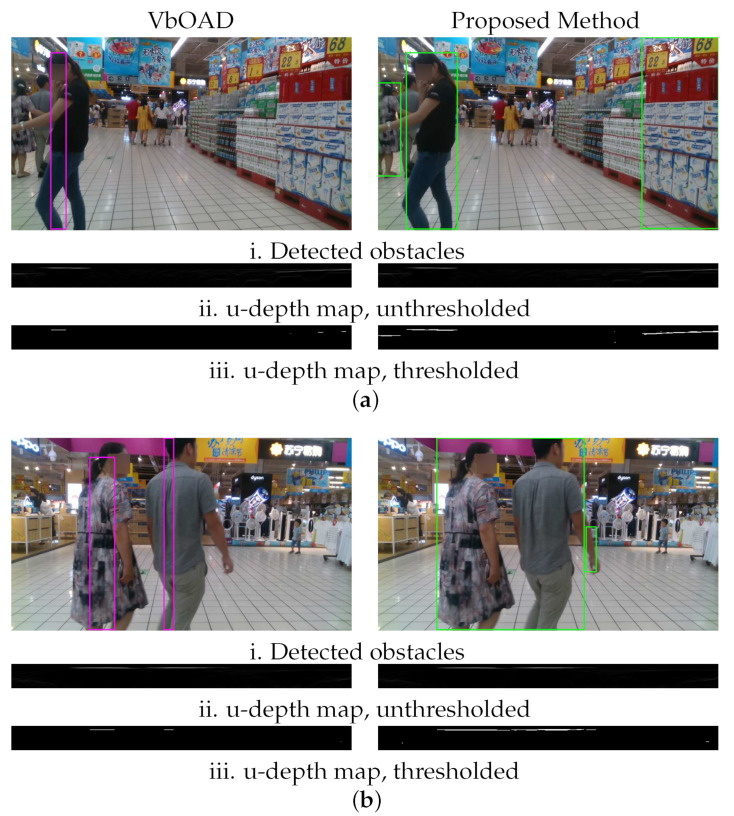
The effectiveness of the proposed dynamic thresholding against fixed thresholding as proposed in VbOAD [28] on the market sequence of the Open LORIS-Scene data set [29]. (**a**) Example 1. (**b**) Example 2.

We compared the effect of the proposed dynamic thresholding with that of fixed thresholding as proposed in VbOAD. The edges of the white patches on the u-depth map do not become bright when an obstacle has a bent shape, and fixed thresholding may cut the edges of such white patches that are not as bright, irrespective of their position and size. We show this phenomenon in Figure 23 with two examples using the $Set_{5}$ data from Table 1. Figure 23a presents the first example, where Figure 23a(i) shows the comparison of detected obstacles on the same snapshots and Figure 23a(ii) shows the corresponding unthresholded u-depth map. Figure 23a(iii) shows the corresponding thresholded u-depth map. VbOAD generated incorrect dimensions of the obstacle in the u-depth map with a fixed threshold value, as shown in the first column of Figure 23a(iii). The thresholding generated an erroneous result due to the bent shape of the leg of the standing lady in Figure 23a(i). The proposed method with dynamic thresholding produced an accurate thresholded u-depth map, as shown in the second column of Figure 23a(iii). Figure 23b shows a similar pattern to that in Figure 23a for the second example. In this example, the thresholding of VbOAD generated an erroneous result, as shown in the first column of Figure 23b(iii), because the obstacles were very close to the camera in this frame, and both sides of the white patches fell below the given fixed threshold value. The proposed dynamic thresholding generated more accurate dimensions in this case, as shown in the second column of Figure 23b(iii).

### 4.5. Experiments with Multiple Dynamic Obstacles ($Set_{4}$)

**Figure 24:**
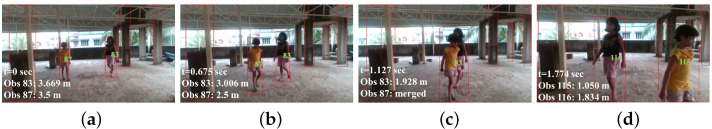
Performance of the proposed tracking algorithm with two moving obstacles along with multiple static obstacles. (**a**–**d**): Time-sampled snapshots with the results using our proposed method.

We tested the proposed method with two dynamic obstacles and multiple static obstacles with the $Set_{4}$ data of Table 1. Two walking girls were considered dynamic obstacles, where they walked at an average velocity of 1 m/s but in different directions. The two dynamic obstacles crossed in proximity. Figure 24 shows the tracking results along with the time stamps and estimated distances of both the detected dynamic obstacles. The proposed method detected the two dynamic obstacles and successfully tracked them, as shown in Figure 24a,b. Then, the proposed method considered two obstacles as a single one when they came into proximity, as shown in Figure 24c, and again considered them two new obstacles as they became farther apart, as shown in Figure 24d.

### 4.6. Indoor Open Sequence ($Set_{5}$)

We have already shown the accuracy and performance of the proposed method on the self-captured simulated data, open video sequences, and multiple self-captured data sequences. This experiment showed the performance of the proposed method on another open data sequence (i.e., $Set_{5}$ in Table 1, the market sequence of the LORIS-Scene data set). The data set environment is a real-world departmental shop with multiple dynamic and static obstacles. Figure 10 shows the details of the tracking results, whereas Figure 10a shows one such snapshot where the system tracked all obstacles as shown with their IDs. Figure 10b shows the corresponding Rviz [35] snapshot, where a dynamic obstacle (a segmented point cloud) is represented as a red rectangular parallelepiped. The rectangular parallelepiped dimensions are annotated, which were used to estimate the state of the dynamic obstacle. It also shows the velocity direction with a yellow arrow (not in the actual scale for better visibility).

### 4.7. Experiments with a Fast-Moving Obstacle ($Set_{4}$)

**Figure 25:**
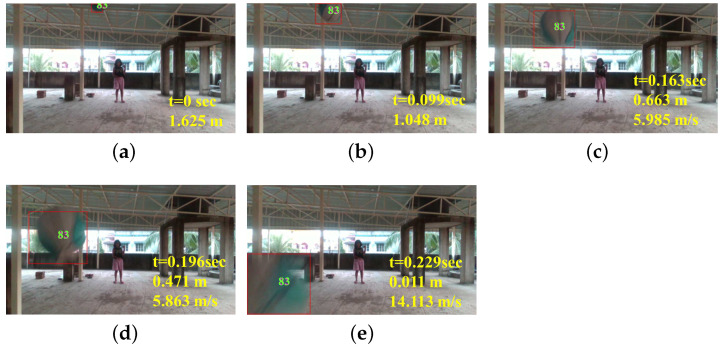
Performance of the proposed tracking algorithm with a very fast-moving obstacle. (**a**–**e**): Time-sampled snapshots with the results using our proposed method.

We test the proposed method with a fast-moving basketball with the $Set_{4}$ data of Table 1, where a basketball was thrown toward the camera at an average speed of around 5 m/s. Figure 25 presents the experiment’s outcome, where each snapshot shows only the estimated dynamic obstacles for better visibility. The time stamps, estimated distances, and estimated velocities are denoted on each snapshot. The algorithm initially failed to detect the ball when it looked small and far from the camera because the proposed dynamic thresholding of the u-depth map gave more priority to nearby objects, even with a small size. Therefore, the proposed thresholding rejected the ball, as it appeared to be very small in size and far away from the camera. However, the proposed thresholding successfully detected the basketball from about 1.625 m away, as shown in Figure 25a, and tracked it until it went out of the FOV. The average estimated velocity was about 7.114 m/s. The basketball was coming toward the camera from afar, where the basketball’s motion was parallel to the camera’s viewing direction. Therefore, the positional change of the obstacle in the u-depth map was relatively low, and the proposed system successfully tracked the ball, but the system may fail to detect obstacles if any obstacle moves quickly from left to right or vice versa.

### 4.8. Execution Time

**Figure 26:**
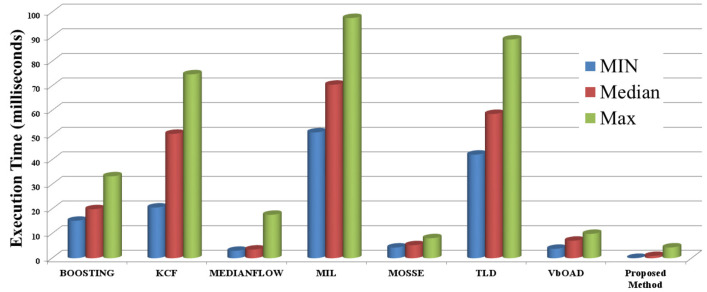
Maximum, median, and minimum execution time comparison for single-obstacle tracking.

Figure 26 shows a comparison of execution times for the experiment presented in Section 4.2.3. We present the minimum, median, and maximum times for all the algorithms, where the time details are shown for a single obstacle tracking with an initialization resolution of the testing obstacle on an RGB image of 174×403. We excluded the initialization time from the plot for all RGB image-based tracking algorithms for better visibility, because these values ranged between 18.5641 and 100.2011 ms and affected the maximum time value. In contrast, the initialization time of the proposed method was 4.3722 ms. We can see from Figure 26 that the execution times for BOOSTING, KCF, MIL, and TLD were very high compared with those of MedianFlow, MOSSE, VbOAD, and the proposed method. The time details of VbOAD were taken from the literature because the source code is not open, and our unoptimized implementation took much longer than the claimed time. Therefore, we restricted our further comparison representation to only be among MedianFlow, MOSSE, and the proposed method for better visibility and understanding.

**Figure 27:**
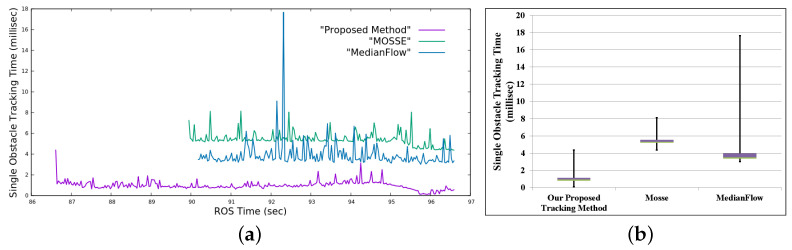
Comparison in execution time for single-obstacle tracking: (**a**) continuous running time and (**b**) the box plot.

Figure 27 presents different representations of execution time among the proposed method, MOSSE, and MedianFlow for the same experiment as that presented in Section 4.2.3. Figure 27a represents the continuous running time comparison, and Figure 27b represents the box plot. The x-axis (ROS Time [46]) of Figure 27a was mapped onto a specific scale for better visibility. The proposed method had a minimum execution time in every case, as shown in Figure 27a. The 75th percentile of the tracking time for a single obstacle in our proposed method was below 1.15 ms, and the maximum tracking time for a single obstacle was 4.37 ms, as shown in Figure 27b. The 75th percentile of the tracking time for a single obstacle in the VbOAD algorithm was below 8 ms, as reported in the literature. Therefore, the proposed method was more than two times faster, and we can conclude that the proposed method can perform @60 Hz in parallel with any real-time SLAM and path planner modules.

## 5. Conclusions

This article presents an obstacle detection and tracking system in dynamic environments using depth images for robotic applications. In this work, we used a u-depth map for detecting obstacles and a restricted v-depth map along with a u-depth map for accurate estimation of the dimensions of obstacles. We introduced a dynamic binary thresholding on the u-depth map to improve the accuracy of obstacle detection and estimation of the obstacle dimensions. We proposed an efficient algorithm to track obstacles under different scenarios, such as indoor or outdoor environments, direct or indirect sunlight, multiple dynamic obstacles moving in multiple directions, obstacles with fast motion, dynamic obstacles of small sizes, and dynamic obstacles dynamically changing their shapes and sizes. The performance of the proposed system was tested using multiple self-captured and open data sequences. The proposed detection and tracking system ran on board @60 Hz. On average, we achieved a 0.6-ms time per obstacle detection and tracking computation and successfully tracked an obstacle at a maximum speed of 5 m/s. The performance of the proposed system was superior to the SoA methods in terms of the accuracy of the obstacle’s state estimation and execution time. Hence, the proposed system can be used for dynamic obstacle detection in mobile robot navigation. The system is limited by the obstacle’s speed and cannot detect a very fast-moving obstacle. We shall focus on multi-sensor fusion to alleviate this problem and detect very fast-moving obstacles.

## Figures and Tables

**Table 1:** Configurations of all experimental data sets.

#	Data Set	Type	Mounted on	Depth Sensor	Image Size and Rate (Hz)	Obstacle Description (Dynamic)
$Set_{1}$	Gazebo	Indoor	Husky	Microsoft	480×640	Single
	Simulation [33]		Robot [39]	Kinect [12]	30	
$Set_{2}$	PTB [1]	Indoor	Fixed or	Microsoft	480×640	Single,
			Handheld	Kinect [12]		Multiple
$Set_{3}$	DepthTrack [22]	Indoor/	Fixed or	Realsense 415	360×640	Single,
		outdoor	Handheld	[13]		Multiple
						Small Obstacle,
						Fast-Moving
$Set_{4}$	Self-Captured	Outdoor,	Handheld	RealSense	480×848	Single,
		Shaded		D435i [13]	60	Multiple,
		Sunlight,				Dynamic Size
		Direct				and Shape,
		Sunlight				Small Obstacle,
						Fast-Moving
$Set_{5}$	OpenLORIS-Scene	Indoor	Wheeled	RealSense	480×848	Single,
	[29]		Robot	D435i [13]	30	Multiple,
						Multiple
						Heights

**Table 2:** Experimental evaluation on PTB [1] and DepthTrack [22] data sets.

Data Set	Sequence	Type	*ACC_avg_*
PTB	bear_front	Indoor	0.952
	child_no1	Indoor	0.934
	face_occ5	Indoor	0.981
	new_ex_occ4	Indoor	0.952
	zcup_move_1	Moving camera	0.913
DepthTrack	ball10_wild	Very Small Obstacle, Direct Sunlight	0.821
	cube03_indoor	Very Small Obstacle Random Motion	0.8521
	duck03_wild	Daylight Condition, Moving Camera	0.921
	hand01_indoor	Very Small Obstacle	0.8195
	human02_indoor	Human Motion	0.948
	pot_indoor	Very High Motion	0.9142
	squirrel_wild	Jerky Motion Moving Camera	0.871
	suitcase_indoor	Indoor	0.9333

## Data Availability

In this manuscript, the employed data sets have been taken with license agreements from the corresponding institutions through proper channels.

## References

[1] Song S., Xiao J. Tracking Revisited Using RGBD Camera: Unified Benchmark and Baselines. Proceedings of the IEEE International Conference on Computer Vision.

[2] Gibbs G., Jia H., Madani I. (2017). Obstacle Detection with Ultrasonic Sensors and Signal Analysis Metrics. Transp. Res. Procedia.

[3] Beltran D., Basañez L. (2013). A Comparison between Active and Passive 3D Vision Sensors: BumblebeeXB3 and Microsoft Kinect. Adv. Intell. Syst. Comput..

[4] Labayrade R., Aubert D., Tarel J. Real time obstacle detection on non flat road geometry through v-disparity representation. Proceedings of the IEEE Intelligent Vehicles Symposium.

[5] Labayrade R., Aubert D. In-vehicle obstacles detection and characterization by stereovision. Proceedings of the 1st International Workshop on In-Vehicle Cognitive Computer Vision Systems.

[6] Oleynikova H., Honegger D., Pollefeys M. Reactive avoidance using embedded stereo vision for MAV flight. Proceedings of the IEEE International Conference on Robotics and Automation.

[7] Bertozzi M., Broggi A., Fascioli A., Nichele S. Stereo vision-based vehicle detection. Proceedings of the IEEE Intelligent Vehicles Symposium 2000 (Cat. No.00TH8511).

[8] Burlacu A., Bostaca S., Hector I., Herghelegiu P., Ivanica G., Moldoveanu A., Caraiman S. Obstacle detection in stereo sequences using multiple representations of the disparity map. Proceedings of the International Conference on System Theory, Control and Computing (ICSTCC).

[9] Song Y., Yao J., Ju Y., Jiang Y., Du K. (2020). Automatic Detection and Classification of Road, Car, and Pedestrian Using Binocular Cameras in Traffic Scenes with a Common Framework. Complexity.

[10] Martinez J.M.S., Ruiz F.E. Stereo-based aerial obstacle detection for the visually impaired. Proceedings of the Workshop on Computer Vision Applications for the Visually Impaired.

[11] Huang H., Hsieh C., Yeh C. (2015). An Indoor Obstacle Detection System Using Depth Information and Region Growth. Sensors.

[12] Zhang Z. (2012). Microsoft kinect sensor and its effect. IEEE Multimed..

[13] Keselman L., Woodfill J., Grunnet-Jepsen A., Bhowmik A. Intel(R) RealSense(TM) Stereoscopic Depth Cameras. Proceedings of the IEEE Conference on Computer Vision and Pattern Recognition Workshops (CVPRW).

[14] Zhu X., Wu X., Xu T., Feng Z., Kittler J. (2020). Complementary Discriminative Correlation Filters Based on Collaborative Representation for Visual Object Tracking. IEEE Trans. Circuits Syst. Video Technol..

[15] Zhu X.F., Wu X.J., Xu T., Feng Z.H., Kittler J. (2021). Robust Visual Object Tracking Via Adaptive Attribute-Aware Discriminative Correlation Filters. IEEE Trans. Multimed..

[16] Xu T., Feng Z., Wu X., Kittler J. (2021). Adaptive Channel Selection for Robust Visual Object Tracking with Discriminative Correlation Filters. Int. J. Comput. Vis..

[17] Hannuna S., Camplani M., Hall J., Mirmehdi M., Damen D., Burghardt T., Paiement A., Tao L. (2019). DS-KCF: A Real-Time Tracker for RGB-D Data. J. Real-Time Image Process..

[18] Henriques J.F., Caseiro R., Martins P., Batista J. (2015). High-Speed Tracking with Kernelized Correlation Filters. IEEE Trans. Pattern Anal. Mach. Intell..

[19] Kart U., Kamarainen J.K., Matas J. How to Make an RGBD Tracker ?. Proceedings of the European Conference on Computer Vision (ECCV) Workshops.

[20] Liu Y., Jing X., Nie J., Gao H., Liu J., Jiang G. (2018). Context-Aware Three-Dimensional Mean-Shift With Occlusion Handling for Robust Object Tracking in RGB-D Videos. IEEE Trans. Multimed..

[21] Qian Y., Yan S., Lukezic A., Kristan M., Kämäräinen J.K., Matas J. DAL: A Deep Depth-Aware Long-term Tracker. Proceedings of the International Conference on Pattern Recognition.

[22] Yan S., Yang J., Käpylä J., Zheng F., Leonardis A., Kämäräinen J.K. DepthTrack: Unveiling the Power of RGBD Tracking. Proceedings of the IEEE/CVF International Conference on Computer Vision (ICCV).

[23] Danelljan M., Bhat G., Khan F., Felsberg M. ATOM: Accurate Tracking by Overlap Maximization. Proceedings of the IEEE Conference on Computer Vision and Pattern Recognition.

[24] Bhat G., Danelljan M., Van Gool L., Timofte R. Learning Discriminative Model Prediction for Tracking. Proceedings of the IEEE/CVF International Conference on Computer Vision (ICCV).

[25] Yang G., Chen F., Wen C., Fang M., Liu Y., Li L. A new algorithm for obstacle segmentation in dynamic environments using a RGB-D sensor. Proceedings of the IEEE International Conference on Real-time Computing and Robotics.

[26] Odelga M., Stegagno P., Bülthoff H. Obstacle detection, tracking and avoidance for a teleoperated UAV. Proceedings of the IEEE International Conference on Robotics and Automation (ICRA).

[27] Luiten J., Fischer T., Leibe B. (2020). Track to reconstruct and reconstruct to track. IEEE Robot. Autom. Lett..

[28] Lin J., Zhu H., Alonso-Mora J. Robust vision-based obstacle avoidance for micro aerial vehicles in dynamic environments. Proceedings of the 2020 IEEE International Conference on Robotics and Automation (ICRA).

[29] Shi X., Li D., Zhao P., Tian Q., Tian Y., Long Q., Zhu C., Song J., Qiao F., Song L. Are We Ready for Service Robots?. The OpenLORIS-Scene Datasets for Lifelong SLAM. In Proceedings of the International Conference on Robotics and Automation (ICRA).

[30] Qin T., Li P., Shen S. (2018). Vins-mono: A robust and versatile monocular visual-inertial state estimator. IEEE Trans. Robot..

[31] Zhang D. Extended Closing Operation in Morphology and Its Application in Image Processing. Proceedings of the International Conference on Information Technology and Computer Science.

[32] Wu K., Otoo E., Suzuki K. (2009). Optimizing two-pass connected-component labeling algorithms. Pattern Anal. Appl..

[33] Koenig N., Howard A. Design and use paradigms for Gazebo, an open-source multi-robot simulator. Proceedings of the IEEE/RSJ International Conference on Intelligent Robots and Systems (IROS) (IEEE Cat. No.04CH37566).

[34] Hartley R., Zisserman A. (2004). Multiple View Geometry in Computer Vision.

[35] Kam H., Lee S.H., Park T., Kim C.H. (2015). RViz: A toolkit for real domain data visualization. Telecommun. Syst..

[36] Rehder J., Nikolic J., Schneider T., Hinzmann T., Siegwart R. Extending kalibr: Calibrating the extrinsics of multiple IMUs and of individual axes. Proceedings of the IEEE International Conference on Robotics and Automation.

[37] Hu M. (1962). Visual pattern recognition by moment invariants. IRE Trans. Inf. Theory.

[38] Koubaa A. (2016). Robot Operating System (ROS): The Complete Reference (Volume 1).

[39] Gariepy R., Mukherjee P., Bovbel P., Ash D. (2019). Husky: Common Packages for the Clearpath Husky. https://github.com/husky/husky.

[40] Schapire R.E. (2013). Explaining Adaboost. Empirical Inference.

[41] Kalal Z., Mikolajczyk K., Matas J. Forward-Backward Error: Automatic Detection of Tracking Failures. 20th International Conference on Pattern Recognition.

[42] Babenko B., Yang M.H., Belongie S. Visual tracking with online Multiple Instance Learning. Proceedings of the IEEE Conference on Computer Vision and Pattern Recognition.

[43] Bolme D., Beveridge J., Draper B., Lui Y. Visual object tracking using adaptive correlation filters. Proceedings of the IEEE Computer Society Conference on Computer Vision and Pattern Recognition.

[44] Kalal Z., Mikolajczyk K., Matas J. (2012). Tracking-Learning-Detection. IEEE Trans. Pattern Anal. Mach. Intell..

[45] Bradski G. (2000). The OpenCV Library. Dr. Dobb’s J. Softw. Tools.

[46] ROS Time: A Class Under ROS Package. http://wiki.ros.org/roscpp/Overview/Time.

